# Noninvasive diagnosis of Alzheimer’s disease: a review of emerging biomarkers and multimodal approaches

**DOI:** 10.3389/fcell.2026.1964029

**Published:** 2026-10-08

**Authors:** Junsheng Zhao, Chenqi Nie, Rong Gao, Zekai Hu, Yajie Lin

**Affiliations:** 1 Bioinformatic Center, Key Laboratory of Artificial Organs and Computational Medicine in Zhejiang Province, Shulan International Medical College, Shulan (Hangzhou) Hospital, Zhejiang Shuren University, Hangzhou, China; 2 State Key Laboratory for Diagnosis and Treatment of Infectious Diseases, National Clinical Research Center for Infectious Diseases, National Medical Center for Infectious Diseases, Collaborative Innovation Center for Diagnosis and Treatment of Infectious Diseases, The First Affiliated Hospital, Zhejiang University School of Medicine, Hangzhou, China

**Keywords:** Alzheimer’s disease, blood-based biomarkers, cognitive and functional phenotype, multimodal assessment, noninvasive biomarkers

## Abstract

Noninvasive biomarkers are expanding access to biological assessment of Alzheimer’s disease (AD). This review evaluates blood-based, neuroimaging, retinal, electrophysiological, digital, and behavioral measures alongside structured cognitive and functional assessment, and examines multimodal and machine-learning approaches for detection, differential diagnosis, prognosis, and treatment monitoring. Plasma phosphorylated tau, particularly p-tau217, currently offers the strongest scalable evidence for identifying amyloid and advanced tau pathology, whereas PET and MRI retain complementary roles in pathological confirmation, regional staging, exclusion of alternative causes, and characterization of atypical phenotypes. Retinal, electrophysiological, speech, sleep, and eye-tracking measures remain promising but require greater standardization and external validation. Across modalities, biomarker trajectories are temporally staggered, assay and platform differences affect interpretation, and pathology–phenotype mapping is modified by disease stage, cognitive reserve, copathology, vascular factors, and population characteristics. We therefore propose a phenotype-anchored, stepped pathway that begins with patient and informant history, objective cognitive and functional assessment, structural imaging, and routine laboratory evaluation; adds validated blood biomarkers when they resolve a defined uncertainty; and reserves CSF or PET for unresolved, atypical, or treatment-relevant cases. Discordant findings should prompt reassessment rather than automatic reclassification. We describe the literature-search and descriptive evidence-categorization approach used to select and categorize the evidence reviewed, and we place the findings within the emerging regulatory context of blood-based testing. Clinical translation will depend on harmonized thresholds, representative prospective cohorts, calibration, independent validation, and evidence that multimodal systems improve decisions and patient outcomes rather than benchmark accuracy alone.

## 1 Introduction

Alzheimer’s disease (AD) represents the most prevalent cause of dementia worldwide, yet its diagnosis has historically relied on clinical assessment of cognitive decline and *postmortem* neuropathological confirmation (Apostolova, 2016; Sen et al., 2017). Over the past decade, a fundamental conceptual shift has transformed the understanding of AD from a discrete clinical entity to a continuous disease spectrum that begins years or even decades before the appearance of overt symptoms (Dubois et al., 2016; Sen et al., 2017). The disease process creates a window of opportunity for early detection and disease-modifying therapy (Weiner et al., 2015; Sen et al., 2017). This paradigm shift has prompted the development and refinement of multiple biomarker modalities, including cerebrospinal fluid (CSF) assays, molecular neuroimaging, structural and functional magnetic resonance imaging (MRI), and, more recently, blood-based and digital measures (Weiner et al., 2015; Sen et al., 2017). A landmark development in the biomarker framework for AD was the introduction of the A/T/N classification system in 2016, which categorizes biomarkers into three binary groups based on the pathophysiological process each measures (Jack et al., 2016; Allegri, 2020). Under this system, “A” denotes β-amyloid pathology, assessed through amyloid positron emission tomography (PET) or CSF Aβ42. “T” indicates tau pathology, measured by CSF phosphorylated tau or tau PET; and “N” represents neurodegeneration or neuronal injury, captured by fluorodeoxyglucose (FDG)-PET, structural MRI, or CSF total tau (Jack et al., 2016; Allegri, 2020). This descriptive scheme is agnostic to the temporal ordering of pathogenic events and does not impose clinical diagnostic labels, making it suitable for categorizing biomarker findings across the entire spectrum from cognitively normal individuals to those with dementia (Jack et al., 2016).

Despite these advances, the transition to a biomarker-based diagnostic paradigm has not been without controversy. Recent proposals, including the Revised Criteria for Diagnosis and Staging of Alzheimer’s Disease, advocate for diagnosing AD solely on the basis of biomarker positivity—amyloid “A” or phosphorylated tau “T1” positivity—without requiring the presence of clinical symptoms (Hazan et al., 2024). However, longitudinal data to establish the lifetime risk of dementia in amyloid-positive and tau-positive individuals are still needed (Hazan et al., 2024). There is also concern that limiting biomarker-based AD diagnosis to amyloid and tau alone may be too narrow, potentially missing other contributing pathologies (Hazan et al., 2024). Atypical variants—including dysexecutive AD, behavioral variant AD, posterior cortical atrophy, and the logopenic variant of primary progressive aphasia—present with predominantly nonmemory symptoms and are frequently misdiagnosed (Jones et al., 2024). Imaging techniques such as MRI, FDG-PET, and tau PET reveal region-specific abnormalities that are less tightly coupled to amyloid deposition (Jones et al., 2024). Structural imaging, preferably with MRI, remains essential in clinical evaluation to rule out reversible causes and increase diagnostic confidence. FDG-PET can provide additional diagnostic specificity when the diagnosis remains uncertain after comprehensive clinical and structural assessment. Amyloid PET, tau PET, and functional MRI have demonstrated clinical utility in specific scenarios, yet their routine use in community practice is still constrained by limited accessibility, high costs, and the need for standardized interpretation criteria (Atri, 2016).

Compared with CSF examination, noninvasive diagnosis for AD avoids invasive lumbar puncture-related risks and enables convenient, repeatable clinical screening and dynamic monitoring. The present review synthesizes recent advances in noninvasive diagnosis of AD, with particular attention to how biological evidence should be interpreted alongside the clinical phenotype. Blood-based, neuroimaging, retinal, electrophysiological, digital, and behavioral markers are reviewed together with structured cognitive and functional assessment. We then examine multimodal strategies that combine molecular specificity with clinically meaningful staging. The emphasis is on scalable approaches that improve diagnostic confidence, differential diagnosis, prognosis, or treatment selection rather than on isolated classification accuracy.

Two organizing principles structure the evidence reviewed below. First, we describe each modality by the molecular and cellular process it reflects—amyloidosis, tau phosphorylation and aggregation, astrogliosis, axonal injury, synaptic degeneration, and vascular or blood-brain barrier dysfunction—rather than by diagnostic performance alone. Second, we distinguish the clinical tasks a result is meant to serve: detection of AD-related pathology, biological diagnosis, clinical diagnosis, staging, prognosis, and treatment eligibility. The same analyte can serve different tasks at different disease stages: p-tau217 can support pathological detection and triage, whereas tau PET topography informs staging and prognosis (Figure 1). This framing makes explicit that a positive biomarker is an input to, not a substitute for, clinical judgment.

**FIGURE 1:**
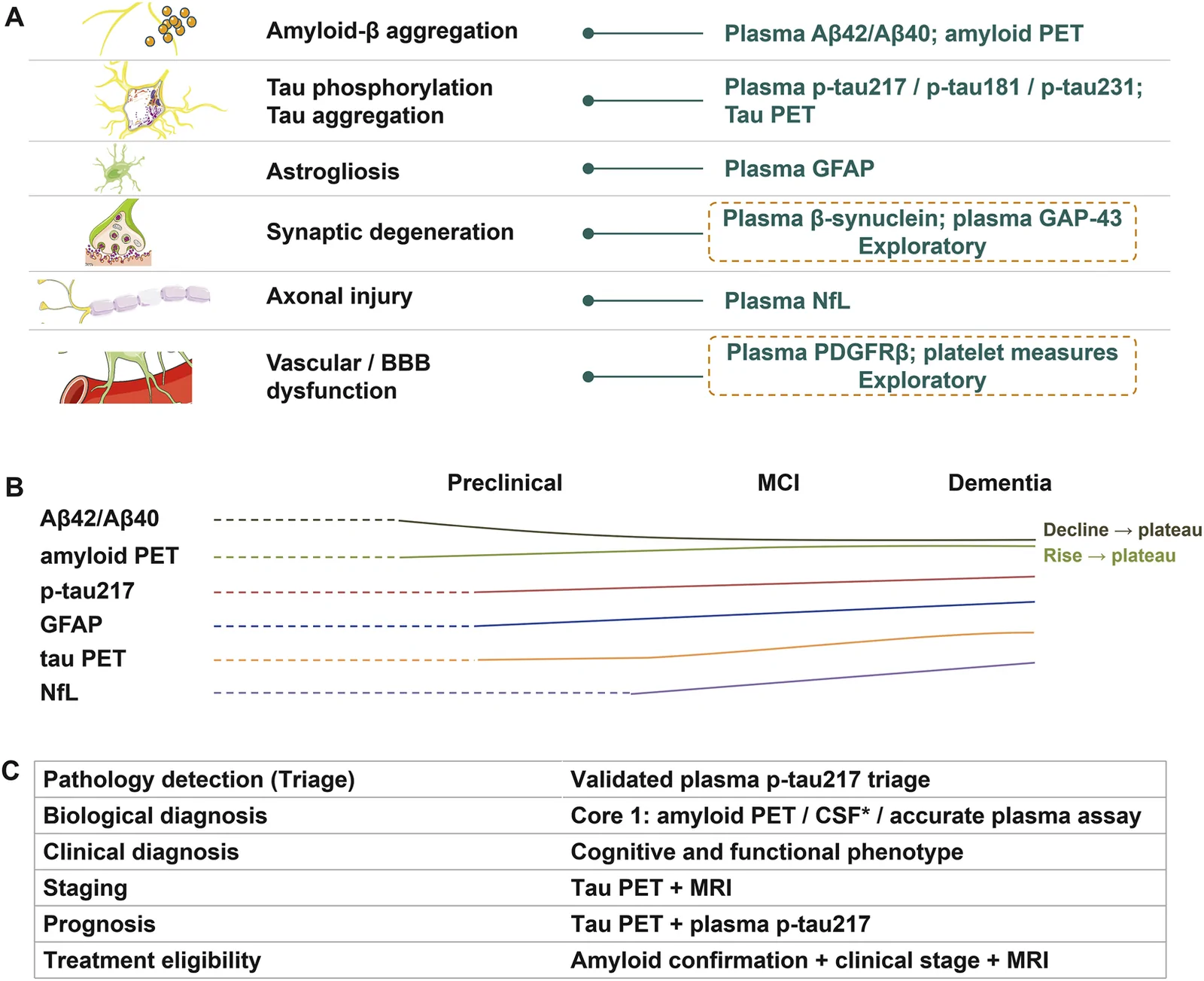
From pathology to clinical use: a mechanistic map of noninvasive Alzheimer’s disease biomarkers. Panel **(A)** links each core pathological process—amyloid-β aggregation, tau phosphorylation and aggregation, astrogliosis, synaptic degeneration, axonal injury, and vascular or blood-brain barrier dysfunction—to the fluid and imaging measures that reflect it (plasma p-tau217, Aβ42/Aβ40, GFAP, NfL, β-synuclein and GAP-43, PDGFRβ and platelet measures, amyloid and tau PET). Measures with exploratory-level evidence are labelled as such. Panel **(B)** sketches the staggered, relative timing of biomarker abnormality across the disease continuum from preclinical to dementia stages; the time axis is ordinal rather than calibrated in years, and plateaus (plasma Aβ42/Aβ40 and amyloid accumulation) are indicated. Panel **(C)** maps each modality to the clinical task it is best suited to serve—detection of AD-related pathology(triage), biological diagnosis, clinical diagnosis, staging, prognosis, or treatment eligibility. CSF* is an established biomarker modality for biological confirmation but is outside this review’s noninvasive scope. This figure is a conceptual synthesis of the evidence reviewed in the text; relative ordering reflects published PET-clock and longitudinal modeling studies.

## 2 Methods

This is a narrative, clinically oriented review. Targeted searches of PubMed/MEDLINE were conducted for combinations of Alzheimer disease, diagnosis, blood biomarker, amyloid beta, phosphorylated tau, MRI, PET, retina, OCT, EEG, speech, gait, digital biomarker, machine learning, multimodal, external validation, and related terms. Landmark articles, systematic reviews, meta-analyses, prospective diagnostic studies, clinical-practice guidelines, consensus statements, regulatory documents, and relevant references cited by these sources were prioritized. Searches were updated through 7 August 2026. Because selection was purposive rather than exhaustive and no preregistered protocol or duplicate screening was used, the article should not be interpreted as a systematic review.

Study selection prioritized multicenter validation studies, head-to-head assay comparisons, systematic reviews and meta-analyses, and prospective longitudinal cohorts; single-cohort exploratory findings were included where they illustrate emerging modalities or failure modes, and are labelled accordingly. Evidence maturity in Table 1 was categorized descriptively for the stated clinical use, based on incorporation into diagnostic criteria, appropriate-use guidance, or standard clinical evaluation and the extent of validation. The categories were defined as follows: criterion-supported indicates incorporation into formal diagnostic criteria, appropriate-use guidance, or standard clinical evaluation; advanced validation indicates replication across independent cohorts or by head-to-head comparison without standardization for routine use; exploratory indicates single-cohort, limited-validation, or protocol-stage evidence. These categories summarize validation status and incorporation into clinical guidance; they do not constitute formal ratings of study quality or certainty of evidence. The seven dimensions discussed in Section 10 provide broader interpretive context for the strengths, limitations, and translational gaps of the evidence, rather than a separate formal grading or scoring framework. Performance figures are reported as published, with population, reference standard, and assay noted in Table 2; values from different studies are not directly comparable. Because this is a narrative rather than systematic review, the search was not exhaustive, no formal risk-of-bias assessment was performed, and the descriptive maturity categories reflect our assessment of the evidence for the stated clinical use.

**TABLE 1:** Comparison of noninvasive modalities for Alzheimer’s disease assessment.

Modality and representative measures	What is measured	Best-supported clinical question	Informative stage window	Key strengths	Main confounders and failure modes	Accessibility, cost and repeatability	Evidence maturity; key references
A. Blood-based measures							
Plasma p-tau217 and p-tau217/Aβ42 ratio	AD-related tau phosphorylation associated with amyloid and tau pathology	Etiological confirmation; triage for amyloid/tau PET; prognosis and staging	Preclinical to dementia	Near-CSF accuracy for amyloid detection; repeatable blood sampling	Assay-specific thresholds; pretest probability; limited rule-in value for the earliest tau deposition	Routine blood draw/low-moderate cost/repeatable; platform availability varies	Advanced validation (Aguillon et al., 2023; Therriault et al., 2023; Ashton et al., 2024; Therriault et al., 2024; Woo et al., 2024; Devanarayan et al., 2025a; Gong et al., 2025; Khalafi et al., 2025; Milà-Alomà et al., 2025; Pan et al., 2025; Shekari et al., 2026; Um et al., 2025; Cogswell et al., 2026; Digma et al., 2026; Kim et al., 2026)
Plasma Aβ42/Aβ40	Relative abundance of circulating amyloid-β peptides	Screening for low amyloid burden	Preclinical; plateaus later	Very early abnormality; scalable sampling	Small dynamic range; platform precision; later plateau; possible fluid-PET discordance	Routine blood draw/low-moderate cost/repeatable	Advanced validation (Liu et al., 2025; Milà-Alomà et al., 2025)
Other phosphorylated tau epitopes and tau fragments (p-tau181, p-tau231, NTA-tau, %p-tau205, MTBR-tau243/eMTBR-tau243)	Epitope-specific tau phosphorylation, truncation, and cortical tau burden	Prognosis and staging; complementary timing information	Preclinical to dementia	Complementary temporal windows; associations with tau PET	Heterogeneous assay biology; limited harmonization, head-to-head testing, and external validation	Research/specialist assay/moderate cost/repeatable	Exploratory (Mielke et al., 2018; Tissot et al., 2022; Lantero-Rodriguez et al., 2023; Therriault et al., 2023; Collij et al., 2026; Lee et al., 2026)
Plasma GFAP	Astrocytic activation	Prognosis and staging; recognition of mixed pathology	Preclinical to dementia	Early change; complements tau measures	Also reflects cerebrovascular and non-AD pathology; may plateau beyond a tau threshold	Routine blood draw/low-moderate cost/repeatable	Advanced validation (Diaz-Galvan et al., 2024; Boerwinkle et al., 2025; Milà-Alomà et al., 2025; Pan et al., 2025; Lockhart et al., 2026)
Plasma NfL	Neuroaxonal injury without AD specificity	Prognosis and staging; repeated monitoring	MCI to dementia; later change	Sensitive to axonal injury; useful for severity monitoring	Affected by non-AD neurodegeneration and vascular injury; becomes abnormal relatively late	Routine blood draw/low-moderate cost/repeatable	Advanced validation, non-specific (Milà-Alomà et al., 2025; Pan et al., 2025; Lockhart et al., 2026)
Synaptic markers (β-synuclein, GAP-43)	Synaptic degeneration and remodeling	Prognosis and staging	Preclinical to dementia	β-synuclein may rise preclinically; longitudinal links to tau, atrophy, and cognition	No established clinical cutoffs; limited assay standardization and implementation evidence	Research assay/moderate cost/repeatable	Exploratory (Oeckl et al., 2023a; Oeckl et al., 2023b; Lan et al., 2025)
Vascular, blood-brain barrier, and haemostatic markers (PDGFRβ, platelet aggregation; SDC4 currently CSF-only)	Blood-brain barrier integrity, endothelial glycocalyx, and haemostatic behavior	Risk stratification; prognosis in mixed pathology	Preclinical to dementia	Captures vascular modulation of downstream injury	Sex-dependent or nonlinear associations; SDC4 lacks plasma validation; no clinical thresholds	Research assay/variable cost/repeatability not established	Exploratory (Ramos-Cejudo et al., 2025; Li et al., 2026; Tarawneh et al., 2026)
B. Neuroimaging							
Structural and diffusion MRI	Atrophy, cortical thickness, vascular injury, and axonal integrity	Differential diagnosis; prognosis and staging	Across the continuum	First-tier anatomy and copathology assessment; no ionizing radiation	Scanner/protocol heterogeneity; vascular confounding; treatment-related pseudo-atrophy; limited molecular specificity	Widely available/moderate-high cost/repeatable	Criterion-supported (Jacobs et al., 2020; Scheijbeler et al., 2023; Kumar et al., 2025; Gong et al., 2026)
Amyloid PET	Fibrillar Aβ plaque burden	Etiological confirmation; treatment eligibility	Preclinical to dementia	Established molecular specificity; standardized visual and quantitative interpretation	Early plateau; limited severity staging; tracer/read variability; requires context for mixed pathology	Specialist centre/high cost/limited repeatability; radioactive tracer	Criterion-supported (Farrell et al., 2024; Trudel et al., 2025; Zamani et al., 2025; Chiotis et al., 2026; Xin et al., 2026; Zhu et al., 2026)
Tau PET	Spatial distribution and burden of fibrillar tau pathology	Etiological confirmation; differential diagnosis; prognosis and staging	Preclinical detection emerging; strongest from MCI onward	Topography aligns with phenotype and decline; adds staging information	Tracer-specific binding and off-target signal; earliest-tau detection floor; visual-read standardization; amyloid status still required for anti-amyloid eligibility	Specialist centre/high cost/limited repeatability; radioactive tracer	Criterion-supported (Cho et al., 2016; Botha et al., 2018; Rubinski et al., 2022; Cho et al., 2023; Rabinovici et al., 2025; Trudel et al., 2025; Xin et al., 2026)
FDG-PET	Regional glucose metabolism and synaptic dysfunction	Differential diagnosis	MCI to dementia	Functional topography; useful when structural assessment does not resolve etiology	Not pathology-specific; affected by disease stage and other neurologic conditions	Specialist centre/high cost/limited repeatability; radioactive tracer	Criterion-supported (Atri, 2016; Rubinski et al., 2020)
Hybrid PET/MR acquisition	Simultaneous molecular, structural, and vascular information	Etiological confirmation in complex cases	MCI to dementia	Single-session coregistration; patient convenience	Very limited availability; high cost; longer scan; retains PET tracer and interpretation limitations	Specialist centre/very high cost/limited repeatability	Exploratory (Schütz et al., 2016)
C. Retinal and ocular measures							
Retinal structural and vascular imaging (OCT, OCT-A, confocal scanning laser ophthalmoscopy)	Retinal thickness, microvasculature, and neurodegenerative change	Screening; repeated monitoring	MCI to dementia; preclinical use under study	Non-contact or minimally burdensome; can leverage eye-care infrastructure	Ocular disease, age, device and acquisition protocol; incomplete normative data	Ophthalmic centre/moderate cost/repeatable	Exploratory (Bader et al., 2024; Gaire et al., 2024)
Hyperspectral and other exploratory retinal imaging	Retinal spectral signatures and putative amyloid-related change	Screening for early pathology	Preclinical under study	Potential molecular sensitivity in an accessible tissue	Prospective protocol still ongoing; retinal/systemic confounding; limited standardization	Research setting/cost uncertain/potentially repeatable	Exploratory (Bader et al., 2024; Gaire et al., 2024)
D. Electrophysiology							
EEG and MEG (spectral slowing, connectivity, event-related responses, epileptiform detection)	Millisecond-scale neural dynamics and network dysfunction	Complementary functional characterization; repeated monitoring	MCI to dementia	High temporal resolution; repeatable; detects occult hyperexcitability	Vigilance, medication, age, comorbidity, acquisition protocol, and weak external validation	Specialist service/moderate cost/repeatable	Exploratory (Moretti, 2015a; Jiao et al., 2023; Costanzo et al., 2024; Nous et al., 2024; Ryoo et al., 2025)
E. Digital and behavioural measures							
Speech and language analysis	Pausing, acoustic features, semantic organization, and language-network behavior	Screening; repeated monitoring	Preclinical to dementia	Low-burden remote capture; repeated measurement	Language, culture, task, device, privacy, and limited biomarker-confirmed external validation	Remote/research setting/low cost/repeatable	Exploratory (Ferrante et al., 2024; Qi et al., 2025; Wang et al., 2025)
Sleep EEG and wearable monitoring	Sleep architecture, oscillatory coupling, and sleep-wake behavior	Prognosis and staging; repeated monitoring	Preclinical to dementia	Home-based longitudinal sampling; links network physiology to cognition	Night-to-night variability; medication and sleep disorders; hardware and preprocessing differences	Home/research setting/low-moderate cost/repeatable	Exploratory (Pulver et al., 2023; Pedersen et al., 2025; Wei et al., 2025; Callow et al., 2026; Constantino et al., 2026)
Digital eye tracking	Oculomotor and visual-cognitive behavior	Screening for cognitive impairment	MCI to dementia	Rapid and noninvasive; potentially deployable in eye clinics	Visual status and device effects; comparator assays may be available only in subsets; no external validation	Eye clinic/research setting/low-moderate cost/repeatable	Exploratory (Qi et al., 2025; Ling et al., 2026)
Olfactory testing, brief clinical signs, and behavioural questionnaires (olfactory bulb volume/discrimination, head-turning sign, Neucop-Q, repetitive negative thinking)	Olfactory function, interview behavior, and psychological risk-related behavior	Screening; risk stratification	Preclinical to dementia	Low burden; compatible with first-line assessment	Non-specific sensory, psychiatric, cultural, and informant effects; mainly cross-sectional evidence	Widely available/low cost/repeatable	Exploratory (Marchant et al., 2020; Daté et al., 2024; Liao et al., 2024)
F. Cognitive and functional assessment							
Brief global screens (MMSE, MoCA)	Phenotypic anchor: global cognitive impairment across multiple domains	Screening	Across the continuum	Rapid case finding; familiar longitudinal benchmarks	Language, education, age, culture, sensory status, ceiling effects, and practice effects	Widely available/low cost/repeatable with practice-effect controls	Criterion-supported (Nasreddine et al., 2005; Li et al., 2023)
Domain-based neuropsychological assessment	Phenotypic anchor: domain pattern, severity, reserve, and atypical presentation	Differential diagnosis; prognosis and staging	MCI to dementia	Resolves atypical phenotypes; links symptoms to functional neuroanatomy	Language and cultural norms; education; sensory status; requires trained personnel	Specialist service/moderate cost/repeatable with limitations	Criterion-supported (Jack et al., 2024; Jones et al., 2024)
Functional and staging instruments and preclinical composites (FAQ, CDR/CDR-SB, PACC)	Phenotypic anchor: functional consequence, global stage, and subtle longitudinal change	Prognosis and staging; repeated monitoring	Preclinical to dementia	Patient/informant relevance; longitudinal sensitivity	Informant quality; intercurrent illness; measurement error; practice effects	Widely available to specialist service/low-moderate cost/repeatable	Criterion-supported (Pfeffer et al., 1982; Morris, 1993; Nasreddine et al., 2005; Donohue et al., 2014; Hazan et al., 2024; Jack et al., 2024)
G. Multimodal models							
Models combining measured modalities	Joint information from molecular pathology, anatomy, physiology, cognition, and function	Etiological confirmation; differential diagnosis; prognosis and staging	Across the continuum	Complementary evidence; can improve stratification over single measures	Missing data, scanner/assay heterogeneity, data leakage, demographic bias, calibration, and interpretability	Research/specialist setting/variable cost/repeatability depends on pipeline	Mixed/study-specific (Yu et al., 2024; Devanarayan et al., 2025b; Karlsson et al., 2025; Kumar et al., 2025; Trudel et al., 2025; Wang et al., 2025; Huang et al., 2026; Tong et al., 2026)
Cross-modal predicted representations (knowledge distillation; joint synthesis-classification)	Predicted, not measured, PET-like or pathology-related representations from MRI	Triage and reduced inference burden	MCI to dementia	MRI-only inference after paired-modality training; tested in external datasets	Predictions are not measured pathology; domain shift, calibration, and interpretability remain unresolved	Research setting/variable cost/repeatable after deployment	Exploratory (Wang et al., 2024; Wang et al., 2026)

Maturity: Criterion-supported = incorporated into formal diagnostic criteria, appropriate-use guidance, or standard clinical evaluation; Advanced validation = replicated across independent cohorts or by head-to-head comparison but not yet standardized for routine use; Exploratory = single-cohort, limited-validation, or protocol-stage evidence. Mixed/study-specific is used for the broad category “Models combining measured modalities”; it indicates that maturity varies across the cited models and should be judged for each study individually. It is not an additional formal maturity category. The category refers to the stated clinical use. These descriptive categories follow the Methods and are not a composite score across the seven interpretive dimensions discussed in Section 10.

Interpretation: Cognitive and functional measures are phenotypic anchors, not AD-specific biological markers. PET, is noninvasive but requires a radioactive tracer and specialist infrastructure. CSF, measures are excluded from the main modality rows; SDC4 is currently supported only in CSF, and requires plasma validation.

Abbreviations: AD, Alzheimer’s disease; Aβ, amyloid-β; BBB, blood-brain barrier; CDR, Clinical Dementia Rating; CDR-SB, CDR, Sum of Boxes; CSF, cerebrospinal fluid; EEG, electroencephalography; FAQ, Functional Activities Questionnaire; FDG, fluorodeoxyglucose; GFAP, glial fibrillary acidic protein; MCI, mild cognitive impairment; MEG, magnetoencephalography; MMSE, Mini-Mental State Examination; MoCA, Montreal Cognitive Assessment; MRI, magnetic resonance imaging; NfL, neurofilament light chain; OCT, optical coherence tomography; OCT-A, OCT, angiography; PACC, Preclinical Alzheimer Cognitive Composite; PDGFRβ, platelet-derived growth factor receptor-β; PET, positron emission tomography; SDC4, syndecan-4.

**TABLE 2:** Representative validation evidence for noninvasive markers of Alzheimer’s disease, organized into clinically validated or advanced-stage evidence (A) and exploratory and digital/AI evidence (B).

Study or cohort	Population and setting	n	Index measure or model	Reference standard or outcome	Design and follow-up	Main reported performance or association	External or multicentre validation	Conclusion the study supports	Principal limitation; Ref
A. Clinically validated or advanced-stage evidence (blood-based and imaging)									
Knight ADRC mixed-dementia cohort	AD, DLB, FTD, PD, and CU research participants	3,232	NULISAseq CNS Panel; plasma p-tau217	Amyloid PET, tau PET, CSF Aβ42/Aβ40, and CDR	Observational; follow-up NR	Amyloid-PET concordance 93.77%; AUC 0.96 for amyloid positivity but 0.81 for clinical AD diagnosis	No	Plasma p-tau217 identifies amyloid pathology more accurately than it establishes a clinical AD diagnosis, supporting phenotype anchoring	Single research centre; no external validation; clinical-diagnosis performance was lower. (Gong et al., 2025)
Systematic review and meta-analysis of 30 studies	Pooled cognitively unimpaired and impaired populations	30 studies	Plasma and CSF p-tau217	Amyloid PET and tau PET	Systematic review; search through August 2024	For amyloid PET: plasma sensitivity/specificity 82%/86%; CSF 79%/91%. Tau-PET sensitivity: plasma 83% vs. CSF 91%	N/A (meta-analysis)	Plasma p-tau217 has strong pooled accuracy for amyloid pathology, with lower sensitivity than CSF for tau pathology	Assay, threshold, population, and stage heterogeneity; performance was better in cognitively impaired participants. (Khalafi et al., 2025)
TRIAD head-to-head study	Dementia-specialist research cohort with amyloid and tau PET	174	Plasma vs. CSF p-tau217, p-tau181, and p-tau231	Amyloid PET positivity	Cross-sectional	p-tau217 AUC: plasma 0.91 vs. CSF 0.94; p-tau181 0.76 vs. 0.87; p-tau231 0.82 vs. 0.95	No	Plasma p-tau217 approached CSF performance for amyloid positivity in this specialty cohort; other plasma epitopes performed less well	Single specialist cohort; no external validation; not a population-screening study. (Therriault et al., 2023)
Cross-continuum biomarker study	Participants across cognitive and biological AD stages	1,275	Plasma p-tau217, GFAP, and NfL	Aβ/tau stage, hippocampal atrophy, and cognitive decline	Observational; follow-up NR	p-tau217 separated A-/T- or A-/T+ from A+/T + Braak III-VI; early A+/T- and Braak I-II stages were identified only among Aβ+ participants; GFAP plateaued and NfL associations were limited	No	The three blood markers carry different staging information; p-tau217 is most specific to advanced AD pathology in this study	Single study; early-stage classification depended on prior Aβ positivity; no external validation. (Pan et al., 2025)
A4 Biological Stage A study	Clinically unimpaired, amyloid-PET-positive, medial-temporal-tau-PET-negative trial participants	222 of 335	Baseline plasma p-tau217 and amyloid PET burden	Tau-PET accumulation, MRI atrophy, and PACC decline	Longitudinal: tau PET 4.2 years; MRI 4.2 years; PACC 5.7 years	Higher baseline p-tau217 and amyloid burden were associated with faster tau accumulation, greater atrophy, and faster cognitive decline	No	Baseline plasma and amyloid measures can stratify risk within very early biological AD.	Selected A4 trial cohort; observational associations do not establish treatment benefit; no external validation. (Digma et al., 2026)
TRIAD, WRAP, and SPIN cohorts	Three observational research cohorts	786	ALZpath Simoa plasma p-tau217	Amyloid/tau PET or CSF-defined AT status	Cross-sectional with longitudinal analysis; follow-up NR	AUC 0.92–0.96 for abnormal amyloid and 0.93–0.97 for tau; annual rise only in Aβ+ participants; three-range approach reduced confirmatory testing by about 80%	Yes	ALZpath p-tau217 showed reproducible multicohort performance and can support a triage strategy	Academic research cohorts and assay-specific thresholds; low-prevalence clinical utility remains unproven. (Ashton et al., 2024)
Automated-platform validation cohort	Clinical cohort across the AD continuum; tau-PET subgroup	262; tau PET 76	Beckman Coulter Access DxI 9,000 p-tau217/Aβ42 ratio	Amyloid PET positivity; tau PET and AD-related cortical atrophy	Cross-sectional; first platform validation	Amyloid-PET AUC 0.943; indeterminate zone 8.0%; strong associations with tau PET across Braak stages and cortical atrophy	No	The automated ratio is promising for amyloid triage and links to downstream tau and atrophy	First validation of this platform; small tau-PET subgroup; no external validation. (Um et al., 2025)
pTau217R regional tau model	Aβ-positive early AD; model and two validation sets	144 model; 98 VS1; 47 VS2	Immunoprecipitation-mass spectrometry pTau217R model	Regional tau PET: MK-6240 in model/VS1; flortaucipir in VS2	Model development plus two validation sets	ROC 0.84–0.95 in VS1 and 0.71–0.88 in cross-tracer VS2; estimated 65% fewer tau-PET scans at 95% sensitivity	Yes	The ratio model may triage regional tau-PET use in selected Aβ-positive early AD.	Selected Aβ-positive samples; lower cross-tracer performance; PET reduction was model-estimated, not a pragmatic outcome. (Devanarayan et al., 2025a)
ADNI amyloid- and tau-PET clock analysis	Longitudinal ADNI research cohort	Amyloid PET 784; tau PET 359; plasma subsets 190/70	Plasma Aβ42/Aβ40, p-tau217, p-tau181, GFAP, and NfL	Modeled amyloid- and tau-PET timelines	Longitudinal generalized additive mixed modeling	Aβ42/Aβ40 became abnormal very early then plateaued; p-tau217 and GFAP changed early on the tau timeline; NfL changed later	No	Plasma analytes show a relative sequence of abnormality that can inform stage-matched interpretation	Modeled rather than prospective clock time; small plasma subsets; abnormality timing varied across six assay platforms. (Milà-Alomà et al., 2025)
ALFA + early-tau study	Cognitively unimpaired participants with PET, MRI, fluid markers, and cognition	97	CSF and plasma p-tau217 and other fluid markers	18F-RO948 tau PET in Braak I/II regions	Cross-sectional plus ROC analysis	Correlation with early tau PET: CSF p-tau217 r = 0.58; plasma r = 0.37. PPV 0.09–0.33, with high NPV	No	Fluid markers can help rule out, but cannot reliably rule in, the earliest tau deposition without PET.	Small cognitively unimpaired cohort; low prevalence constrains PPV; no external validation. (Shekari et al., 2026)
ADNI and A4/LEARN longitudinal comparison	Two independent longitudinal research datasets	292 (ADNI 141; A4/LEARN 151)	Change in tau PET, plasma p-tau217, cortical thickness, and amyloid PET	Change in MMSE, ADAS-Cog13, CDR-SB, and PACC	Longitudinal mixed models and bootstrapping; duration NR	Tau PET, p-tau217, and cortical-thickness change tracked cognitive-change rates; amyloid-PET change did not	Yes	Several noninvasive measures can monitor cognitive-change rates, whereas serial amyloid-PET change is less informative	Observational datasets; follow-up duration NR in terminal manuscript; cortical thickness may be confounded by treatment-related pseudo-atrophy. (Biel et al., 2025)
Consecutive Han Chinese memory-clinic cohort	SCD, MCI, and dementia in a specialist memory clinic	1,008	18F-Florzolotau tau PET	Amyloid PET (cerebral amyloid pathology)	Prospective consecutive recruitment; cross-sectional diagnostic-impact assessment, 2020–2025	Sensitivity 97.8% and specificity 95.4% vs. amyloid PET; adding tau PET changed diagnosis in 10.0% after amyloid PET and raised confidence from 68.6% to 84.2%	No	Tau PET showed high concordance with amyloid pathology and added diagnostic information after amyloid PET.	Amyloid PET, not independent AD diagnosis or autopsy, was the reference; single centre/ethnicity; tracer and visual-read generalizability are uncertain. (Xin et al., 2026)
BioFINDER-2	Amyloid-positive participants; serial-imaging subgroup	761; longitudinal imaging 322	Baseline and longitudinal 18F-RO948 tau PET vs. 3T structural MRI	Cognitive decline	Longitudinal imaging and mediation models; follow-up NR	Baseline tau PET explained 54.0%–94.0% of cognitive-decline variance; longitudinal tau-PET/MRI mediation 2.0%–15.0%; simulated tau-slope reduction changed trajectories only marginally	No	Baseline tau burden was more prognostic than MRI or longitudinal change in this amyloid-positive cohort	Single amyloid-positive cohort; only 322 had serial imaging; simulation does not establish a causal treatment effect. (Singleton et al., 2025)
TRIAD, ADNI, and HABS-HD biological staging	Cognitively unimpaired and MCI participants across three cohorts	492	A + T2MTL+, A + T2MOD+, and A + T2HIGH + tau-PET staging	Clinical progression and cognitive decline	Longitudinal; mean follow-up 2.49 years	Biological stage predicted progression better than amyloid PET, tau PET, or demographics alone; late tau stage in MCI: 83% dementia by 4 years; A + T2HIGH+: 50% progressed by 2.36 years	Yes	Regional tau staging adds prognostic information beyond binary PET status	Observational research cohorts; prediction depends on PET-defined stages and requires pragmatic clinical validation. (Trudel et al., 2025)
Study or cohort	Population and setting	n	Index measure or model	Reference standard or outcome	Design and follow-up	Main reported performance or association	External or multicentre validation	Conclusion the study supports	Principal limitation; Ref
B. Exploratory and digital/AI evidence									
BeyeOMARKER	Planned eye-clinic cohort, age≥50 years, without prior dementia diagnosis	700 planned; 150 imaging subgroup	Plasma p-tau217 and hyperspectral retinal imaging; OCT/OCT-A and widefield imaging secondary	Aβ- and tau-PET visual reads; cognitive decline	Prospective observational protocol, April 2024-March 2027; about 2 years follow-up	NR - no results reported	NR - no results reported	The protocol tests feasibility and validation design; it does not yet support a performance conclusion	Ongoing protocol; all diagnostic-performance fields remain unreported. (Bader et al., 2024)
Digital eye-tracking study	Older adults: CU and cognitively impaired	276 (119 CU; 157 CI); plasma subgroup 154	Composite digital eye-tracking panel	CI vs. CU classification; plasma, PET, and MRI comparators in subsets	Cross-sectional	Eye-tracking AUC 0.865; plasma Aβ42/Aβ40 0.699, p-tau217 0.769, p-tau217/Aβ42 0.801, GFAP 0.804, NfL 0.826	No	Eye tracking can screen cognitive impairment, but the study does not establish AD pathology or cost-effectiveness	Eye-tracking AUC used all 276 participants, whereas plasma AUCs used only 154; not a same-sample head-to-head comparison. (Ling et al., 2026)
Scoping review of digital and behavioural AI studies	431 studies across motor, neurocognitive, eye-tracking, and speech domains	431 studies; 86 AI models	Digital and behavioural AI models	Study-specific endpoints	Scoping review	Mean AUC: 0.887 for 21 AD models and 0.821 for 45 MCI models; only 2 studies externally validated and 3 assessed calibration	N/A (review)	High benchmark discrimination coexists with weak external validation and calibration	Marked task, dataset, and reporting heterogeneity; pooled benchmark performance does not establish real-world utility. (Qi et al., 2025)
Cookie-theft speech and serum-biomarker study	Probable AD, aMCI, and CU participants; amyloid PET in a subset	1,223 (238 AD; 461 aMCI; 524 CU)	PSD, APOE, GFAP/p-tau217, demographics; XGBoost/logistic models	Cognitive-impairment classification and Aβ status	Cross-sectional machine-learning study	AUC 0.928 for cognitive impairment and 0.845 for Aβ status; PSD, p-tau217, and GFAP showed two-stage change	No	Speech contributes complementary phenotypic information when combined with blood and demographic measures	Amyloid PET was available only in a subset; single dataset; external validation and calibration were not reported. (Wang et al., 2025)
Coupled sleep-rhythm study	AD dementia, MCI due to AD, and cognitively normal participants	93 (33 AD; 38 MCI; 22 CN)	SO-theta-burst and SO-spindle coupling from sleep EEG	Clinical group, amyloid/tau burden, and 2-year cognitive decline	Longitudinal; 2 years	Coupling frequency declined from CN to MCI and became further misaligned in dementia; disruption predicted accelerated 2-year decline; CSF/MRI integration improved prediction	No	Coupled sleep rhythms are candidate monitoring and prognostic markers rather than stand-alone etiological tests	Small single cohort; no external validation; multimodal gain included invasive CSF. (Wei et al., 2025)
Head-turning sign and Neucop-Q	CN, MCI, dementia, and psychiatric-disease participants in a memory-clinic setting	155	Head-turning sign and three-question Neucop-Q	Amyloid and tau PET	Cross-sectional	For amyloid PET: HTS specificity 0.930 and PPV 0.870; impaired awareness and news knowledge NPV 0.750 and 0.725	No	Simple interview signs may support first-line screening in memory clinics	Modest mixed sample; cross-sectional; non-specific signs cannot replace biomarker or clinical evaluation. (Daté et al., 2024)
E2AD knowledge distillation and joint synthesis-classification	ADNI development/internal testing; AIBL and NACC external datasets	NR	Paired MRI-PET training; MRI-only inference; PET-like representation synthesis	Classification performance in internal and external datasets	Model development plus external validation; follow-up NR	MRI-only inference was feasible after paired-modality training; external validation was reported; terminal manuscript did not report performance values	Yes	Cross-modal models can reduce inference-time modality burden, but their PET-like representations remain predictions	Sample size and performance values NR in terminal manuscript; predicted representations are not measured pathology and may shift across domains. (Wang et al., 2024; Wang et al., 2026)

Scope: The table retains 21 representative evidence rows, separated into clinically validated or advanced-stage evidence (panel A) and exploratory or digital/AI, evidence (panel B) to distinguish clinical readiness. Redundant single-assay comparisons (Therriault et al., 2024) and advanced-tau thresholding (Woo et al., 2024) were omitted to preserve modality breadth. Standard cognitive-tool validation is summarized in Table 1; EEG/MEG, classifier studies were not included here because the terminal manuscript did not provide sufficiently complete population, reference-standard, and external-validation fields.

NR: Not reported in the terminal manuscript; this does not imply that the original article did not report the field. For BeyeOMARKER, NR, explicitly means that the cited publication is an ongoing protocol with no results reported.

Validation: Yes denotes testing across independent cohorts, datasets, or prespecified validation sets; No denotes a single cohort or centre; N/A is used for evidence syntheses. Performance values should not be compared across rows without considering population, assay, reference standard, and prevalence.

Abbreviations: AD, Alzheimer’s disease; ADAS-Cog13, 13-item Alzheimer’s Disease Assessment Scale-Cognitive Subscale; ADNI, Alzheimer’s Disease Neuroimaging Initiative; AIBL, Australian Imaging, Biomarkers and Lifestyle study; aMCI, amnestic mild cognitive impairment; AUC, area under the receiver-operating characteristic curve; CDR, Clinical Dementia Rating; CDR-SB, CDR, Sum of Boxes; CI, cognitively impaired; CN/CU, cognitively normal/unimpaired; CSF, cerebrospinal fluid; DLB, dementia with Lewy bodies; FTD, frontotemporal dementia; GFAP, glial fibrillary acidic protein; HABS-HD, Health and Aging Brain Study-Health Disparities; HTS, head-turning sign; MCI, mild cognitive impairment; MMSE, Mini-Mental State Examination; MRI, magnetic resonance imaging; NACC, National Alzheimer’s Coordinating Center; NfL, neurofilament light chain; OCT-A, optical coherence tomography angiography; PACC, Preclinical Alzheimer Cognitive Composite; PD, Parkinson’s disease; PET, positron emission tomography; PPV/NPV, positive/negative predictive value; PSD, percentage of silence duration; SO, slow oscillation; SUVR, standardized uptake value ratio; WRAP, Wisconsin Registry for Alzheimer’s Prevention.

## 3 Blood-based biomarkers for AD diagnosis

The development of blood-based biomarkers for AD has accelerated substantially since 2015, driven by the need for scalable, minimally invasive, and accessible diagnostic tools that can complement or, in some cases, replace CSF analysis and positron emission tomography (PET) imaging (Nabers et al., 2018; Fink et al., 2020). Among the various analytes investigated, phosphorylated tau species, particularly p-tau217, have emerged as the most promising candidates due to their strong associations with core AD pathology and their ability to distinguish AD from other neurodegenerative conditions (Schindler et al., 2024; Gong et al., 2025). Throughout this section, biomarker-class evidence is distinguished from assay-specific clinical use, and diagnosis is separated from staging, prognosis, and treatment selection (Hansson et al., 2023).

Plasma p-tau217 demonstrates diagnostic performance that approaches or matches that of CSF biomarkers. A systematic review and meta-analysis of 30 studies reported that plasma p-tau217 achieved 82% sensitivity for detecting amyloid PET positivity and 86% specificity, while CSF p-tau217 showed 79% sensitivity and 91% specificity for the same outcome (Khalafi et al., 2025). Predictive accuracy was higher among cognitively impaired than cognitively unimpaired individuals, and plasma p-tau217 was less sensitive than CSF for detecting tau PET positivity (83% vs. 91%) (Khalafi et al., 2025). In a head-to-head comparison within the TRIAD cohort, plasma p-tau217 yielded an area under the curve (AUC) of 0.91 for identifying amyloid PET positivity, a value statistically indistinguishable from the CSF p-tau217 AUC of 0.94, whereas plasma p-tau181 (AUC 0.76) and p-tau231 (AUC 0.82) performed consistently worse than their CSF counterparts (AUC 0.87 and 0.95, respectively) (Therriault et al., 2023). These findings suggest that the increased accessibility of plasma p-tau217 is not offset by a meaningful loss of diagnostic accuracy, at least for detecting amyloid pathology. In a cohort of 1,275 participants across the cognitive spectrum, plasma p-tau217 effectively separated amyloid-negative individuals (A–T–/A–T+) from those with advanced tau pathology (A + T + Braak III–VI), whereas earlier stages (A + T–/A + T + Braak I–II) were identified only among amyloid-positive individuals (Pan et al., 2025). Probability-based staging models in a single cohort have proposed a candidate therapeutic window for p-tau217 (approximately 1.9–5.1 pg/mL on that assay), within which individuals were modeled as most likely to benefit from disease-modifying therapies (Kim et al., 2026). These model-derived bounds are assay-specific and require prospective treatment-response validation before they can guide therapy selection. In addition, plasma p-tau217 predicts subsequent brain pathology and cognitive decline in autosomal dominant AD. In presenilin-1 E280A mutation carriers, baseline p-tau217 levels were associated with amyloid PET burden, tau PET burden, and memory performance measured 7.61 years later (Aguillon et al., 2023). In the A4 study, among clinically unimpaired individuals in Biological Stage A (amyloid PET positive, medial temporal tau PET negative), higher baseline plasma p-tau217 and higher baseline amyloid PET burden were each associated with faster tau PET accumulation, greater atrophy, and more rapid cognitive decline over follow-up (Digma et al., 2026). Multiple commercial assays for plasma p-tau217 have been validated and show comparable performance. The ALZpath Simoa assay achieved AUCs of 0.92–0.96 for detecting abnormal amyloid and 0.93–0.97 for detecting tau pathology across three independent cohorts, with longitudinal increases observed only in amyloid-positive individuals (Ashton et al., 2024). A direct comparison of the ALZpath and Janssen p-tau217 assays in 294 participants found that both assays were strongly associated with amyloid and tau PET, both identified AD versus other neurodegenerative diseases (AUC 0.95 and 0.96, respectively), and annual changes in both assays correlated with annual changes in tau PET (Therriault et al., 2024). The p-tau217/Aβ42 ratio measured on an automated platform (Beckman Coulter) showed the highest diagnostic accuracy for amyloid PET positivity (AUC 0.943) and the smallest indeterminate zone (8.0%), and it correlated strongly with tau PET across Braak stages and with AD-related cortical atrophy (Um et al., 2025). Direct cross-assay comparisons require caution, however: platforms differ in antibodies, calibrators, and concentration scales, so numerical values—and even AUCs—are not directly interchangeable. Head-to-head multicenter comparisons show broadly high but measurably different performance across p-tau217 assays (Warmenhoven et al., 2025), a round-robin exercise by the Alzheimer’s Association Global Biomarker Standardization Consortium revealed limited commutability of reference materials across platforms (Ashton et al., 2025), and recent multicenter data confirm clinically relevant platform differences (Lv et al., 2026). Clinical cutoffs must therefore be assay-specific; most platforms implement two-cutoff rule-in/rule-out strategies separated by an indeterminate zone whose width varies by assay and by kidney function, body mass index, and anemia (Um et al., 2025; Yun et al., 2026). Triage strategies built on validated blood tests reduced confirmatory PET or CSF testing by roughly two-thirds to four-fifths in studied cohorts (Ashton et al., 2024; Devanarayan et al., 2025a), although pragmatic trials must still demonstrate downstream-test reduction in routine care.

Beyond p-tau217, other phosphorylated tau epitopes and tau fragments provide complementary information. Plasma p-tau181 increases with clinical severity and is associated with both amyloid and tau PET, though its diagnostic accuracy is generally lower than that of p-tau217 (Mielke et al., 2018). Plasma p-tau231 reflects earlier stages of tau pathology and shows distinct temporal dynamics compared with p-tau181 and p-tau217 (Tissot et al., 2022). N-terminal tau fragments (NTA-tau), measured with a novel immunoassay, are more closely associated with tau PET than with amyloid PET or neurodegeneration, and plasma NTA-tau increases over time only in cognitively impaired amyloid-β-positive individuals (Lantero-Rodriguez et al., 2023). The phosphorylation occupancy of tau at threonine 217 (%p-tau217) changes earliest in the disease cascade, increasing just before amyloid PET positivity, while %p-tau205 and MTBR-tau243 align with tau PET positivity onset, and plasma eMTBR-tau243 tracks cortical tau burden in later stages (Collij et al., 2026). CSF and plasma tau species such as eMTBR-tau243, MTBR/243–254, p-tau205, and p-tau217 all demonstrate comparable correlations with tau PET, suggesting that multiple fluid analytes could serve as surrogate markers for PET-based tau assessment (Lee et al., 2026).

Plasma p-tau217 also identifies individuals with advanced tau accumulation. Using adjusted cutoffs, plasma p-tau217 (ALZpath) separated Braak V+ tau PET-positive individuals from other amyloid-positive individuals with an AUC of 0.93–0.97 across two independent cohorts (Woo et al., 2024). A p-tau217 ratio-based model predicted regional tau levels up to a standardized uptake value ratio of 2 in multiple brain regions, with ROC areas of 0.84–0.95 in the primary validation set and 0.71–0.88 in a second validation set acquired with a different tracer, and reduced the need for tau PET scans by 65% while maintaining 95% sensitivity (Devanarayan et al., 2025a). These capabilities are clinically important for stratifying patients for treatments and clinical trials, particularly as tau-targeting therapies become available.

The temporal ordering of plasma biomarker changes has been characterized using amyloid and tau PET clocks. In analyses from the AD Neuroimaging Initiative, plasma Aβ42/Aβ40 became abnormal very early in both amyloid and tau PET timelines, preceding established PET thresholds, and its levels subsequently plateaued (Milà-Alomà et al., 2025). Plasma p-tau217, p-tau181, GFAP, and NfL increased throughout the modeled disease progression, with p-tau217 and GFAP becoming abnormal early in the tau PET timeline (Milà-Alomà et al., 2025). These findings suggest that plasma Aβ42/Aβ40 may be useful for identifying individuals with very low levels of amyloid pathology, whereas p-tau, GFAP, and NfL may be more useful for staging disease progression (Milà-Alomà et al., 2025). In the Mayo Clinic Study of Aging and ADNI, the timing of plasma p-tau217 progression explained a similar degree of variability in cognitive decline as amyloid PET, supporting its utility as a marker of disease progression (Cogswell et al., 2026).

Glial fibrillary acidic protein (GFAP), a marker of astrocytic activation, provides complementary information to p-tau217. In the DLB continuum, abnormal plasma GFAP levels were found at the prodromal stage of mild cognitive impairment with Lewy bodies in association with increased amyloid PET, while abnormal p-tau181 and NfL appeared only at the dementia stage (Diaz-Galvan et al., 2024). In Down syndrome, plasma GFAP partially mediated the relationship between amyloid PET and tau PET (15.3%) and between amyloid PET and plasma p-tau217 (42.1%), indicating that astrogliosis is a key component in the advancement of preclinical AD pathophysiology (Boerwinkle et al., 2025). Plasma GFAP levels plateau beyond a certain tau threshold, while subsequent tau progression was observed only among individuals with high GFAP—an association consistent with, but not proof of, a facilitating role of astrocytic activation in tau propagation (Pan et al., 2025). In a diverse community-based cohort, GFAP was positively associated with white matter hyperintensities, indicating that GFAP reflects both AD-related and cerebrovascular pathology (Lockhart et al., 2026).

Neurofilament light chain (NfL), a marker of axonal injury, shows weaker associations with core AD pathology. In the cohort of 1,275 participants, plasma NfL exhibited limited associations with Aβ/tau pathology, AD-specific hippocampal atrophy, and cognitive decline, leading to the conclusion that NfL is inadequate as a specific neuronal injury biomarker for AD (Pan et al., 2025). However, in the context of mixed pathology, NfL is positively associated with white matter hyperintensities and white matter free water, suggesting that it may be more sensitive to cerebrovascular disease than to AD-specific neurodegeneration (Lockhart et al., 2026). Plasma NfL becomes abnormal later in the disease course, after amyloid and tau PET positivity, and its levels increase throughout progression, making it a potential marker of disease severity rather than early detection (Milà-Alomà et al., 2025).

Synaptic biomarkers have emerged as a new class of blood-based analytes. Plasma β-synuclein, a marker of synaptic degeneration, is already elevated in preclinical AD (amyloid PET-positive cognitively unimpaired individuals) and shows further increases in MCI and AD dementia (Oeckl et al., 2023b). Plasma β-synuclein levels correlate with amyloid and tau PET, are associated with temporal cortical thinning, and can discriminate amyloid PET-positive from amyloid PET-negative individuals in memory clinic patients (Oeckl et al., 2023a). Growth-associated protein 43 (GAP-43), previously identified as a synaptic candidate in CSF, has been measured in plasma and shown to correlate with CSF GAP-43, with both baseline and longitudinal synaptic PET, and with greater tau aggregation, faster brain atrophy, and accelerated cognitive decline, particularly among cognitively unimpaired individuals (Lan et al., 2025).

Vascular and blood-brain barrier biomarkers add another dimension to blood-based AD assessment. Plasma platelet-derived growth factor receptor-β (PDGFRβ) is associated with the CSF/plasma albumin ratio, a measure of BBB integrity, and lower PDGFRβ levels are associated with higher Aβ and tau PET burden, thinner cortical thickness, worse cognitive scores, and more rapid decline (Li et al., 2026). The relationships between plasma tau biomarkers and longitudinal neurodegeneration and cognitive decline are stronger in males with low PDGFRβ levels, suggesting that BBB damage modulates the impact of tau pathology on downstream outcomes (Li et al., 2026). Syndecan-4 (SDC4), a brain endothelial glycocalyx protein measured in CSF, is elevated in the earliest preclinical stages of AD, predicts more rapid progression of brain amyloid and tau, and is among the top proteins in predicting pseudo-time models of AD progression (Tarawneh et al., 2026). While these markers require further validation in plasma, they highlight the potential of blood-based biomarkers to capture vascular contributions to AD. Platelet-related measures have also been linked to AD pathology. In middle-aged participants from the Framingham Heart Study, platelet aggregation response exhibited a nonlinear association with AD pathology. Among those in the lowest tertile of adenosine diphosphate response, platelet aggregation was positively associated with increased amyloid in the precuneus and tau in multiple AD-vulnerable regions, as well as with an MRI-based cortical thickness signature of AD risk (Ramos-Cejudo et al., 2025). These findings support further investigation of platelet-mediated mechanisms in AD pathogenesis and suggest that hemostatic markers may have value as early indicators of AD risk.

Interpretation of plasma biomarkers must also account for systemic confounders. Chronic kidney disease elevates plasma p-tau217 independent of brain amyloid: quantitative modelling shows an inverse association between estimated glomerular filtration rate (eGFR) and p-tau217 concentration, large enough to move individual patients across diagnostic cutoffs (Bornhorst et al., 2025), and meta-analytic evidence confirms that kidney function is associated with several AD-related fluid biomarkers (Arslan et al., 2025; Lin et al., 2026). Phosphorylated-to-total tau ratios partially mitigate this confounding (Janelidze et al., 2023), and assay cutoffs perform differently across strata of kidney function, body mass index, and hemoglobin (Yun et al., 2026). Routine laboratory evaluation—including renal function—therefore belongs in the same diagnostic workup as the blood biomarker itself.

Other conditions can mimic or distort AD biomarker profiles. Plasma GFAP and NfL rise in cerebrovascular disease, GFAP abnormalities appear early in the Lewy body continuum, and NfL elevations occur across many axonal injury disorders, so a positive result is not specific to AD and similar biomarker abnormalities characterize other neurodegenerative diseases (Diaz-Galvan et al., 2024; Lockhart et al., 2026). Pre-analytical factors also matter: plasma p-tau217, Aβ42, and related analytes show diurnal variation, with morning nadirs and afternoon-to-evening peaks whose magnitude for p-tau217 has been compared with the disease-related increase observed over 1 year in amyloid-positive individuals (della Monica et al., 2024), although a controlled morning-versus-evening sampling study found no significant diurnal change for p-tau species while CSF and plasma Aβ42/Aβ40 were higher in the evening (Orduña Dolado et al., 2024). Fasting status likewise influences several analytes, with significant fasting-versus-non-fasting differences reported for p-tau217, NfL, and amyloid ratios in a conference-reported study (Schuder et al., 2025). Together these observations argue for standardized sampling conditions and for reporting comorbidities, renal function, and sampling time alongside biomarker results.

Beyond standard venous sampling, minimally invasive collection strategies are extending the reach of blood-based testing. Finger-prick dried blood spot sampling measured p-tau217, GFAP, and NfL with high concordance to venous plasma, and dried-spot p-tau217 identified CSF-defined AD changes with approximately 86% accuracy in a multicenter study of 337 participants that included self-collected samples (Huber et al., 2026); remote capillary self-sampling correlated with cognition in community-dwelling older adults (Corbett et al., 2026). Neuronal-enriched exosomes isolated from blood are under investigation as biomarker carriers, although meta-analytic evidence remains preliminary and assay standardization is lacking (Zhang et al., 2023). Salivary and urinary analytes are at an earlier stage: salivary p-tau has not consistently associated with AD biomarker status (Gleerup et al., 2025), and urinary studies remain small pilots of exosomal or protein markers (Seol et al., 2020; Sun et al., 2020). Urine-based assessment of renal function is nonetheless relevant to the eGFR adjustment described above. These approaches may improve access and scalability but require analytical and clinical validation before routine use.

Regulatory milestones now shape the clinical context for blood-based testing. In May 2025, the U.S. Food and Drug Administration (FDA) cleared the Lumipulse G pTau217/β-Amyloid 1–42 Plasma Ratio (Fujirebio Diagnostics) through the 510(k) pathway—the first blood-based *in vitro* diagnostic authorized to aid detection of amyloid pathology in adults aged 55 years or older with signs and symptoms of cognitive decline. In the pivotal multicenter study of 499 cognitively impaired adults, 91.7% of ratio-positive individuals had amyloid plaques by PET or CSF, 97.3% of ratio-negative individuals were amyloid-negative, and fewer than 20% of results were indeterminate. The clearance explicitly positions the assay as an adjunct to, not a replacement for, clinical evaluation, and it is not authorized for screening or stand-alone diagnosis. Professional society guidelines converge on similarly cautious use of blood biomarkers within specialized care (Schindler et al., 2024; Palmqvist et al., 2025), and commentators caution about unintended consequences of rapid adoption (Whitson et al., 2026). Regulatory status continues to evolve beyond this single clearance, and the episode illustrates the central theme of this review: even a cleared assay requires interpretation alongside phenotype, pretest probability, comorbidities, and assay-specific thresholds.

The feasibility of implementing blood-based AD tests in diverse, community-based populations has been demonstrated. In a study enrolling 859 participants in St. Louis, 20.6% of whom self-identified as Black or African American, the blood test was well accepted, although it was perceived more positively by White and highly educated individuals (Li et al., 2023). Cognitive impairment screens showed only moderate performance in this diverse population, underscoring the need for blood biomarkers to improve diagnostic accuracy across demographic groups. Universal cutoffs may not generalize, however: longitudinal biomarker trajectories differ by race (Xiong et al., 2022), and assay cutoffs are modified by kidney function, body mass index, and anemia (Yun et al., 2026); population-specific validation is therefore required before broad deployment. The concordance between plasma p-tau217 and amyloid PET status reached 93.77% in a large mixed-dementia cohort using the NULISAseq platform, where plasma p-tau217 achieved an AUC of 0.96 for amyloid positivity but 0.81 for a clinical diagnosis of AD (Gong et al., 2025).

Despite these advances, several challenges remain. Discordance between CSF and PET amyloid biomarkers can occur in certain patients, as illustrated by a case of an APP p.K687Q mutation carrier with reduced CSF Aβ42/Aβ40 but negative florbetapir PET, attributed to potential mechanisms including polymorphic Aβ fibrils lacking ligand binding sites or contributions from cerebral amyloid angiopathy (Liu et al., 2025). Such cases highlight that no single biomarker is infallible and that multimodal assessment may be necessary in ambiguous situations. In addition, the accuracy of fluid biomarkers for detecting tau PET positivity in cognitively unimpaired individuals with very early tau deposition remains limited, with positive predictive values as low as 0.09–0.33 for Braak I/II positivity (Shekari et al., 2026). In the ALFA + cohort, CSF and plasma p-tau217 showed the strongest correlations with early Braak I/II tau PET signal among the fluid biomarkers tested (CSF r = 0.58; plasma r = 0.37), but positive predictive values for the earliest stages of tau accumulation were low. Negative predictive values were nonetheless high, so fluid biomarkers can help rule out early tau pathology, whereas PET remains necessary to confirm it in preclinical populations (Shekari et al., 2026).

## 4 Neuroimaging biomarkers: PET and MRI

PET and MRI provide complementary *in vivo* measures of AD pathology (Mainta et al., 2018).

Amyloid PET has been central to clinical trials of anti-Aβ therapies, where it is used for participant selection, target engagement monitoring, and treatment efficacy assessment (Ten Kate et al., 2018; Chiotis et al., 2026). The updated appropriate use criteria from the Alzheimer’s Association and the Society of Nuclear Medicine and Molecular Imaging have defined seven clinical scenarios in which amyloid PET is considered appropriate, including early-onset cognitive impairment, atypical clinical presentations, and mild cognitive impairment with early age of onset (Rabinovici et al., 2025). However, amyloid PET has limited ability to stage disease severity or predict imminent clinical decline within the timeframe of pre-dementia trials, as amyloid accumulation plateaus early in the disease course and shows weak associations with concurrent cognitive deterioration (Ten Kate et al., 2018; Biel et al., 2025).

Tau PET is an established imaging modality that captures the spatial distribution and progression of neurofibrillary tangle pathology, which correlates more closely with cognitive decline than amyloid burden (Cho et al., 2016; Aschenbrenner et al., 2018; Digma et al., 2019). Multiple tau tracers, including [18F]flortaucipir, [18F]MK-6240, [18F]RO948, and [18F]Florzolotau, have been developed and validated (Kreisl et al., 2022; Lin et al., 2023; Shekari et al., 2026). [18F]MK-6240 recapitulates Braak staging across the AD continuum and shows domain-specific associations with memory, executive function, and language performance in amyloid-positive individuals (Kreisl et al., 2022). [18F]RO948 tau PET detected early tau pathology in individuals with low to moderate amyloid load. Visual reading methods for tau PET, such as the regional scoring system developed for [18F]Florzolotau, demonstrate high inter-reader reliability and significant correlations with global SUVR and clinical dementia severity (Lin et al., 2023).

The diagnostic performance of tau PET has been systematically evaluated in large memory clinic cohorts. In a study of 1,008 consecutive Han Chinese individuals with cognitive impairment, tau PET showed high concordance with amyloid PET as the reference standard for cerebral amyloid pathology (sensitivity 97.8%, 95% CI 96.4–98.7; specificity 95.4%, 95% CI 92.0–97.4) (Xin et al., 2026). Because amyloid PET is itself an imperfect reference standard—detecting fibrillar plaques but not soluble Aβ species and unable to adjudicate non-AD pathologies—concordance with amyloid PET does not prove that a test captures every pathological process it claims to measure. Tau PET provided incremental diagnostic value beyond amyloid PET alone. After a diagnosis based on amyloid PET results, the subsequent addition of tau PET information led to a diagnostic change in 10.0% of patients, and tau PET significantly improved diagnostic confidence from 68.6% to 84.2%, particularly within non-AD diseases (Xin et al., 2026). A probabilistic framework analyzing tau PET predictive values found that tau PET shows high positive and negative predictive values for clinicopathological AD, with a positive tau PET following positive amyloid PET substantially increasing the probability of clinicopathological AD, especially in older adults (van Tol et al., 2026).

Tau PET consistently outperforms other biomarkers in predicting cognitive decline. In the BioFINDER-2 and ADNI cohorts, baseline tau-PET and a composite cognitive score were the strongest independent predictors of cognitive decline over 2 years in patients with amnestic mild cognitive impairment or mild dementia, with cortical thickness and neurofilament light providing only additional information (Smith et al., 2023). Longitudinal analyses from the BioFINDER-2 study, including 761 amyloid-positive individuals, revealed that baseline and longitudinal tau-PET showed stronger associations with cognitive decline than structural MRI, with baseline tau-PET explaining 54.0%–94.0% of the variance in cognitive decline, while longitudinal tau-PET and MRI pathways contributed only modest mediation effects of 2.0%–15.0% (Singleton et al., 2025). These findings emphasize the primacy of baseline tau aggregates for prognostics and treatment considerations in AD (Singleton et al., 2025). Notably, simulated reductions of the tau-PET accumulation slope produced only marginal changes in cognitive trajectories, a finding with direct implications for tau-targeting therapies. Baseline tau-PET also predicts subsequent brain atrophy. In early symptomatic AD patients, global tau-PET signal, but not amyloid-PET, predicted the rate of subsequent atrophy independent of baseline cortical thickness, and the specific distribution of tau-PET signal was a strong indicator of the topography of future atrophy at the single-patient level (La Joie et al., 2020). Regional tau binding in the superior temporal gyrus predicts subsequent atrophy in inferomedial temporal regions, and voxelwise tau binding correlates with future cortical atrophy in superior temporal, parietal, and frontal association cortices (Lagarde et al., 2022).

The relationship between tau and neurodegeneration is also evident in white matter. Cortical tau accumulation is associated with subsequent atrophy in connected white matter regions, with elevated baseline tau-PET in temporal regions associated with lower baseline white matter volume in adjacent regions (Pescoller et al., 2025). Faster tau-PET increase over time was significantly linked to accelerated volume loss in connected white matter regions, and baseline white matter volume did not predict subsequent tau-PET rates of change, suggesting a unidirectional relationship between fibrillar tau and subsequent white matter degeneration (Pescoller et al., 2025). This tau-associated axonal degeneration may contribute to disease progression, and diffusion MRI-derived axonal density indices have demonstrated superior sensitivity in tracking disease progression compared to CSF and PET biomarkers, matching these biomarkers in predicting future cognitive and functional decline (Gong et al., 2026).

Tau PET spatial patterns suggest potential utility in differentiating AD subtypes. Atrophy-defined subtypes of AD, including typical, limbic-predominant, hippocampal-sparing, and mild atrophy, show distinct tau PET patterns. Hippocampal-sparing and typical patients exhibit the greatest neocortical tau load, while limbic-predominant patients show particularly high entorhinal tau (Ossenkoppele et al., 2020). Behavioral/dysexecutive AD is characterized by frontal cortical tau aggregation in medial prefrontal, anterior cingulate, and frontal insular cortices, distinct from amnestic AD, and tau-PET uptake in these regions best differentiates between the two syndromes (Therriault et al., 2021). Nonamnestic AD variants, including posterior cortical atrophy and the logopenic variant of primary progressive aphasia, show syndrome-specific tau-PET binding patterns with stronger effect sizes than MRI atrophy measures (Nasrallah et al., 2018). Early-onset AD shows a parietal-predominant tau-PET pattern, with higher tau burden observed in younger and female participants, and tau-PET mediates the relationship between amyloid-PET and cognitive impairment (Cho et al., 2023). Sex and APOE ε4 carrier status also influence pathologic burden, with female early-onset AD patients showing greater amyloid and tau PET burdens than males, and female APOE ε4 non-carriers having greater amyloid PET burdens and greater gray matter atrophy than female ε4 carriers (Nemes et al., 2023).

The clinical utility of tau PET extends beyond AD diagnosis to include the identification of non-AD tauopathies and clarification of complex cases. In patients clinically diagnosed with non-AD disorders, tau PET led to a significantly higher rate of diagnostic change compared with amyloid PET, and the confidence gained from tau PET was significantly greater than that from amyloid PET alone within non-AD diseases (Xin et al., 2026). The authors nonetheless emphasize that amyloid status remains essential for determining eligibility for anti-amyloid therapies and that amyloid PET cannot be omitted in that context (Xin et al., 2026). Tau-negative amnestic dementia masquerading as AD dementia has been described, where patients with clinical AD features and hippocampal atrophy were amyloid-positive but tau-negative, highlighting the need to establish both amyloid and tau positivity before enrolling patients in trials of disease-modifying therapies (Botha et al., 2018).

Quantification methods for PET imaging continue to evolve to improve sensitivity and standardization. MRI-free pipelines for tau PET processing have been developed that produce SUVRs highly correlated with MRI-based methods, with similar estimates of cross-sectional and longitudinal differences between amyloid-negative and amyloid-positive cognitively unimpaired individuals, even in early focal regions within the medial temporal lobe (Landau et al., 2023; Carlson et al., 2024). The TauIQ algorithm, a canonical image-based approach, calculates global tau load and substantially outperforms SUVR approaches in longitudinal analyses, providing a greater than 2.2-fold increase in power for detecting tau deposition in MCI and a greater than 3.2-fold increase in cognitively normal individuals (Whittington et al., 2021). The petBrain pipeline leverages deep learning-based segmentation for standardized quantification of amyloid, tau, and neurodegeneration biomarkers, demonstrating strong concordance with ADNI databases and good agreement with CSF and plasma biomarkers, clinical status, and cognitive performance (Coupé et al., 2025).

The interplay between amyloid and tau pathology is complex and age- and sex-dependent. Younger age is related to faster amyloid-related tau accumulation in men, and women reach the amyloid PET-inferred tauopathy transition at lower amyloid levels, with these patient-centered thresholds predicting both earlier onset and accelerated cognitive decline (Zhu et al., 2026). Aβ spatial extent, measured as the percentage of neocortex with elevated Pittsburgh Compound-B, improves detection of Aβ below traditional PET thresholds and shows stronger associations with cognition and tau proliferation than Aβ level alone, suggesting that the spatial distribution of amyloid may be more sensitive to Aβ′s role in preclinical AD than its magnitude (Farrell et al., 2024). Predictive modeling has identified that Aβ in the rostral middle frontal, medial orbitofrontal, and striatal regions best predicts entorhinal tau levels, future hippocampal volume, and cognitive decline, indicating their potential as early biomarkers in AD prediction models (Zamani et al., 2025).

Myelin levels modulate regional vulnerability to tau pathology. Higher myelinated cortical regions show lower tau-PET uptake, and higher fiber-tract myelin levels attenuate the association between functional connectivity and longitudinal tau-PET spreading, suggesting that myelination may confer resistance against tau accumulation (Rubinski et al., 2022). Tau pathology uniquely affects sulcal depths, with tau-related cortical thinning predominantly occurring in sulcal regions, and *postmortem* histology confirming preferential tau accumulation in sulcal depths, potentially due to stronger connectivity between these regions and the hippocampus across adulthood (Maboudian et al., 2026).

The integration of amyloid and tau PET within the ATN (amyloid, tau, neurodegeneration) framework has enabled more precise biological staging of AD. In the Alzheimer’s Association biological staging framework, cognitively unimpaired individuals in the A + T2MTL+, A + T2MOD+, and A + T2HIGH + stages were at significantly higher risk of clinical progression compared to non-AD cognitively unimpaired individuals, and biological AD staging demonstrated superior accuracy in predicting clinical progression compared to amyloid-PET status, tau-PET status, and demographic information alone (Trudel et al., 2025). All tau-PET-positive individuals showed a significantly faster rate of cognitive decline than non-AD controls, with the A + T2HIGH + stage showing the steepest rate of decline. Among individuals with MCI, an advanced tau stage was associated with an 83% likelihood of developing dementia over 4 years, and the A + T2HIGH + group reached 50% progression to all-cause dementia at 2.36 years (Trudel et al., 2025).

Structural MRI provides reliable markers of neurodegeneration, including hippocampal atrophy, cortical thinning, and whole-brain atrophy rates, which are useful for tracking disease progression and have the potential to serve as outcome measures in clinical trials (Promteangtrong et al., 2015; Ten Kate et al., 2018). Atrophy measures derived from structural MRI can improve the identification of subjects at risk of imminent decline and play a role in subject inclusion for pre-dementia AD trials (Ten Kate et al., 2018). The combination of MRI with PET imaging in hybrid PET/MR systems allows comprehensive evaluation in a single imaging session, offering increased patient and referrer convenience (Schütz et al., 2016; Mainta et al., 2018). In a pilot study of simultaneous amyloid PET/MRI, the combined evaluation resulted in 31%, 45%, and 24% of mild cognitive impairment subjects being categorized as MCI-unlikely, intermediate likelihood, and high likelihood due to AD, respectively, and 88% of patients or caregivers reported a gain of convenience compared to separate PET and MR imaging sessions (Schütz et al., 2016).

Vascular health and mixed pathologies significantly impact neurodegeneration and AD biomarker interpretation. Cardiovascular and metabolic conditions are associated with greater neurodegeneration in AD signature regions but not with amyloid or entorhinal tau deposition, and structural equation models show that vascular health has a significant direct and indirect impact on neurodegeneration but not on amyloid (Vemuri et al., 2017). White matter hyperintensities, infarcts, hippocampal volumes, and cortical thicknesses can be combined into a quantitative MRI measure that correlates with neurofibrillary tangles, neuronal loss, atrophy, and infarcts at *postmortem*, and predicts conversion to MCI and clinical AD among healthy controls (Brickman et al., 2018). The co-occurrence of AD pathology and vascular brain injury is common, especially among the oldest old, and the cognitive profile of mixed AD and vascular cognitive impairment is dominated by AD, with microinfarcts eluding *in vivo* detection but exerting adverse effects on cognitive function (Chui and Ramirez-Gomez, 2015).

Racial differences in longitudinal AD biomarkers have been observed, with Black participants showing slower decline in CSF Aβ42/Aβ40 and slower accumulation of amyloid on PET compared to White participants, as well as smaller annual increases in CSF total tau and phosphorylated tau181 (Xiong et al., 2022). These differences highlight the importance of considering sociocultural factors and apolipoprotein E genotype in biomarker interpretation and clinical trial design. The PREVENT-AD cohort, which enrolled cognitively normal older adults with a family history of sporadic AD, has shared extensive longitudinal data including multimodal MRI, amyloid and tau PET, plasma biomarkers, and magnetoencephalography, providing a valuable resource for accelerating AD research (Villeneuve et al., 2025).

Systematic comparisons of ATN biomarkers for monitoring longitudinal cognitive changes have revealed that tau-PET, plasma p-tau217, and cortical thickness changes accurately track change rates in cognitive measures, while amyloid-PET change rates are not linked to cognitive changes (Biel et al., 2025). In MCI patients, FDG-PET hypermetabolism is associated with higher tau-PET at low amyloid-PET levels, suggesting a compensatory metabolic response that is accompanied by episodic memory impairment (Rubinski et al., 2020). Regional THK5351 accumulations, which bind both tau and monoamine oxidase-B, correlate with neuropsychological test scores as well as or better than FDG-PET, providing additional utility in estimating AD progression (Im et al., 2023).

Multimodal normative modeling using deep learning-based frameworks has been applied to analyze individual-level variation across ATN imaging biomarkers, revealing greater intra-group heterogeneity in abnormality patterns at more severe clinical stages of AD (Kumar et al., 2025). Individual-level disease severity indices calculated from the spatial extent and magnitude of abnormal deviations across ATN are associated with worse cognitive function and increased risk of disease progression (Kumar et al., 2025).

## 5 Retinal and ocular biomarkers in AD

The retina, as an accessible extension of the central nervous system, is being investigated as a target for noninvasive AD diagnosis. Pathological hallmarks of AD, including amyloid β-protein deposits and abnormal tau protein isoforms, have been identified in the retinas of both AD patients and animal models (Gaire et al., 2024). Structural and functional vascular abnormalities—such as reduced blood flow, vascular Aβ deposition, and blood-retinal barrier damage—along with inflammation and neurodegeneration, have also been described in retinas of individuals with mild cognitive impairment and AD dementia (Gaire et al., 2024). Histological, biochemical, and clinical studies have demonstrated that the nature and severity of AD pathologies in the retina and brain correspond, with proteomics analysis revealing a similar pattern of dysregulated proteins and biological pathways in both tissues, including enhanced inflammatory and neurodegenerative processes, impaired oxidative phosphorylation, and mitochondrial dysfunction (Gaire et al., 2024).

Investigational imaging technologies can now detect AD-specific amyloid deposits, vasculopathy, and neurodegeneration in the retina of living AD patients, suggesting alterations at different disease stages and links to brain pathology(Gaire et al., 2024). Current and exploratory ophthalmic imaging modalities, such as optical coherence tomography (OCT), OCT-angiography, confocal scanning laser ophthalmoscopy, and hyperspectral imaging, may offer promise in the clinical assessment of AD (Bader et al., 2024; Gaire et al., 2024). The BeyeOMARKER study, a prospective, observational, longitudinal cohort study, is designed to evaluate the performance of blood- and eye-based biomarkers for early AD detection in an eye clinic setting (Bader et al., 2024). This study aims to include 700 individuals aged 50 years or older without prior dementia diagnosis, who will undergo blood collection for plasma p-tau217 levels and brief cognitive screening at the eye clinic (Bader et al., 2024). A subset of 150 participants—100 plasma p-tau217 positive individuals and 50 matched negative controls—will additionally undergo Aβ-PET and tau-PET, MRI, retinal imaging including hyperspectral imaging (primary), widefield imaging, OCT and OCT-Angiography (secondary), and cognitive and cortical vision assessments (Bader et al., 2024) The primary outcomes include the performance of plasma p-tau217 and hyperspectral retinal imaging to detect AD pathology, with Aβ- and tau-PET visual read as the reference standard, and to detect cognitive decline over an initial follow-up of approximately 2 years (Bader et al., 2024).

Beyond structural and molecular retinal imaging, digital eye-tracking markers have shown high diagnostic performance. In a study comparing digital eye-tracking tests with plasma biomarkers, the eye-tracking panel exhibited an area under the curve (AUC) of 0.865 in classifying cognitively impaired individuals from cognitively unimpaired individuals, outperforming plasma Aβ42/40 (AUC = 0.699), p-Tau217 (AUC = 0.769), p-Tau217/Aβ42 (AUC = 0.801), glial fibrillary acidic protein (GFAP; AUC = 0.804), and neurofilament light chain (NfL; AUC = 0.826) (Ling et al., 2026). These findings demonstrate that digital eye-tracking markers can serve as a valid and novel tool for screening cognitive impairment in older adults, potentially offering an accessible complement to, rather than a replacement for, pathology-specific blood biomarkers (Ling et al., 2026). The comparison was not performed in the same sample for all measures, and the study does not establish detection of AD pathology itself.

Despite these promising advances, several challenges remain before retinal and ocular biomarkers can be integrated into routine clinical practice. Further research is needed to deepen understanding of AD’s impact on the retina and its progression, and future studies require replication in larger and diverse cohorts with confirmed AD biomarkers and standardized retinal imaging techniques (Gaire et al., 2024). Standardization of imaging protocols, validation across different populations, and establishment of normative data for retinal changes across the aging spectrum will be critical steps toward clinical translation.

## 6 Electrophysiological biomarkers: EEG and MEG

EEG and MEG provide noninvasive measures of neural dysfunction with millisecond temporal resolution. The most reproducible AD-associated pattern is spectral slowing, with increased delta/theta activity, reduced posterior alpha/beta activity, and a lower peak alpha frequency (Cassani et al., 2018; Babiloni et al., 2021; Jiao et al., 2023; Scheijbeler et al., 2023). These changes are detectable across the MCI–dementia continuum and show associations with amyloid burden and cognitive decline. However, their magnitude is influenced by vigilance, medication, age, acquisition protocol, and comorbidity (Moretti D. V., 2015; Scheijbeler et al., 2023; Ryoo et al., 2025).

Connectivity and task-based measures may add information beyond spectral power. AD has been associated with less efficient functional networks, altered cross-frequency coupling, and impaired event-related responses during memory and attention paradigms (Gomez et al., 2016; Li et al., 2020; Casagrande et al., 2023; Simfukwe et al., 2023; Costanzo et al., 2024; Aoki et al., 2026). Prolonged EEG or MEG can also identify subclinical epileptiform activity and network hyperexcitability in a subset of amyloid-positive individuals (Nous et al., 2024). These findings are mechanistically relevant, but they do not yet constitute a disease-specific signature.

Automated classifiers based on entropy, connectivity, spectrograms, or graph neural networks frequently report high discrimination between AD and healthy controls (Tsai et al., 2015; Fan et al., 2018; Lopes et al., 2023; Xu et al., 2025; Zhang et al., 2025). Their clinical value is less certain because many studies use small, highly selected cohorts, non-independent feature selection, and limited external validation. Performance against common mimics—including Lewy body disease, vascular cognitive impairment, depression, and frontotemporal degeneration—is substantially less established (Nardone et al., 2018; Coronel-Oliveros et al., 2024).

Accordingly, electrophysiology is best regarded as a complementary functional modality rather than a first-line etiological test. Its near-term roles include characterizing network dysfunction, detecting occult epileptiform activity, and providing repeatable outcome measures in longitudinal studies or trials. Clinical translation requires harmonized acquisition and preprocessing, pre-specified thresholds, multicenter validation, and demonstration that EEG or MEG improves decisions beyond cognitive assessment, blood biomarkers, and structural imaging (Moretti V. D., 2015; Casula et al., 2022; Lin et al., 2025; Ryoo et al., 2025).

## 7 Digital and behavioral biomarkers: speech and sleep

Digital and behavioral data can complement molecular and neuroimaging measures. These data can often be collected noninvasively, repeatedly, and at low cost. Relevant modalities include speech analysis, sleep electroencephalography (EEG), eye tracking, olfactory testing, clinical signs, and behavioral questionnaires. These measures have been associated with AD pathology and cognitive decline. They may therefore support screening, monitoring, and risk stratification. A scoping review of 431 studies identified motor activity, neurocognitive tests, eye tracking, and speech analysis as major research areas. Classical machine learning models dominated the artificial intelligence (AI) literature. Among 21 AD-focused models, the mean area under the curve (AUC) was 0.887. However, only two studies included external validation, and three assessed model calibration (Qi et al., 2025). Models targeting mild cognitive impairment showed a lower mean AUC (0.821, n = 45 models) than AD-focused models (Qi et al., 2025). These limitations indicate that the field remains methodologically immature.

Speech and language represent one of the most accessible behavioral domains for AD assessment. A large-scale study of 1,223 participants—including 238 with probable AD, 461 with amnestic mild cognitive impairment (aMCI), and 524 cognitively unimpaired (CU) individuals—analyzed speech recordings from the “cookie-theft” picture description task alongside serum biomarkers, with amyloid PET imaging in a subset of participants (Wang et al., 2025). The percentage of silence duration (PSD), a speech-derived digital biomarker, correlated with cognitive level, serum glial fibrillary acidic protein (GFAP), neurofilament light chain (NFL), phosphorylated tau 217 (p-Tau217), and regional amyloid deposition (Wang et al., 2025). Notably, PSD, p-Tau217, and GFAP exhibited a two-stage change pattern across different stages of Aβ deposition, suggesting that speech markers capture both early and progressive pathological transitions (Wang et al., 2025). A machine learning model incorporating PSD, APOE genotype, GFAP, and demographic information achieved an AUC of 0.928 for cognitive impairment diagnosis, while an Aβ status classification model combining PSD, APOE, p-Tau217, and demographic data yielded an AUC of 0.845 (Wang et al., 2025). These results demonstrate that combining speech digital markers with serum biomarkers provides a low-burden, noninvasive diagnostic approach that aligns with underlying AD pathology (health-economic evaluation has not yet been performed).

Beyond simple acoustic features, multivariate word-property analysis of verbal fluency tasks can reveal disease-specific semantic memory patterns that standard valid response counts fail to capture. In a study comparing AD patients to those with behavioral variant frontotemporal dementia (bvFTD) and healthy controls, word frequency, granularity, and neighborhood density yielded robust group- and subject-level discrimination only in AD, while also predicting executive function outcomes (Ferrante et al., 2024). These word properties predicted disease-specific cortical thickness patterns and default-mode and salience network hypoconnectivity, as well as EEG beta hypoconnectivity, exclusively in AD (Ferrante et al., 2024). Thus, automated word-property analysis of fluency tasks can boost AD characterization by revealing neurocognitive markers linked to underlying neural circuit dysfunction.

Sleep provides another rich behavioral data stream with strong biological links to AD pathophysiology. The precise temporal coupling of sleep oscillations—slow oscillations (SOs), theta bursts, and spindles—is essential for memory consolidation, and disruption of this coupling has been reported in AD. In a study of 93 individuals spanning AD dementia, MCI due to AD, and cognitively normal controls, the frequency of SO-theta burst coupling and SO-spindle coupling decreased from normal cognition to MCI, and further misalignment occurred from MCI to AD dementia (Wei et al., 2025). The APOE ε4 allele and elevated amyloid and tau burden were associated with coupled sleep rhythm disruption, while hippocampal and medial prefrontal cortex atrophy were linked to disruption of SO-theta burst coupling and SO-spindle coupling, respectively (Wei et al., 2025). Critically, coupled sleep rhythm disruption predicted accelerated cognitive decline over a 2-year follow-up, and integrating sleep EEG with CSF and MRI biomarkers enhanced predictive ability for AD progression (Wei et al., 2025). These findings position sleep oscillatory coupling as a monitoring and interventional target.

Single-channel sleep EEG, suitable for at-home wearable applications, can also capture memory-associated neural circuit integrity. In 205 aging adults, SW-TB (slow wave-theta burst) and SW-SP (slow wave-spindle) coupling precision was mapped to amyloid positivity, cognitive impairment, and CSF AD biomarkers (Pulver et al., 2023). Cognitively unimpaired, amyloid-positive individuals demonstrated lower precision in both SW-TB and SW-SP coupling compared to amyloid-negative individuals, and significant correlations were found between oscillatory event coupling and CSF Aβ42/Aβ40, phosphorylated tau-181, and total-tau (Pulver et al., 2023). Longitudinal sleep monitoring over approximately 3.5 years in older adults with and without AD pathology revealed that EEG spectral power measures (delta, theta, alpha) demonstrated minimal longitudinal change, whereas self-reported sleep metrics and EEG-derived parameters such as sleep efficiency showed greater variability (Pedersen et al., 2025). This characterization of sleep parameter variability offers important guidance for designing future longitudinal studies targeting sleep and neurodegeneration.

The relationship between sleep disruption and AD pathology may be mediated in part by the glymphatic system, which clears brain waste products during sleep. In a study of 75 individuals, putative glymphatic metrics—including the diffusion tensor imaging along the perivascular space (DTI-ALPS) index and blood oxygen level-dependent signal coupled to CSF signal (BOLD-CSF coupling)—were reduced in AD compared to controls, alongside disrupted SO-spindle coupling (Liu et al., 2026). Lower global BOLD-CSF coupling correlated with misaligned SO-theta burst coupling, and reduced DTI-ALPS was associated with misaligned SO-spindle coupling (Liu et al., 2026). Mediation analysis suggested that the association between SO-spindle misalignment and cognitive decline operates partly through DTI-ALPS, and combining putative glymphatic and sleep EEG metrics effectively predicted AD progression (Liu et al., 2026). These findings suggest that glymphatic clearance and coupled sleep rhythms are jointly associated with AD-related cognitive decline.

Preclinical evidence further supports a causal role for microglia in amyloid-associated sleep loss. In APP/PS1 mice, amyloid plaques caused NREM sleep loss that did not worsen with increased plaque burden, while aging reduced REM sleep and eliminated sleep rebound (Constantino et al., 2026). Amyloid pathology was associated with cortical hyperexcitability, network desynchrony, and microglial expansion extending beyond plaque-bearing regions into thalamocortical and white matter networks governing sleep-wake dynamics. Remarkably, microglial depletion restored more than 2 h of sleep per day without altering amyloid burden, positioning microglia as a causal, reversible driver of amyloid-associated sleep loss and supporting sleep and EEG-based metrics as sensitive biomarkers of presymptomatic AD (Constantino et al., 2026).

The interaction between sleep, physical activity, and AD pathology is moderated by genetic risk and amyloid status. In 120 cognitively unimpaired older adults, greater wake after sleep onset (WASO) was associated with higher tau burden in Braak I-II subregions among APOE ε4 carriers and Aβ-positive individuals, while total volume of physical activity showed opposing associations with tau burden depending on Aβ status (Callow et al., 2026). These risk-dependent associations highlight the importance of stratification in lifestyle-based intervention studies.

Supervised tensor factorization of sleep EEG data from the National Sleep Research Resource identified discriminative sleep patterns in AD, including patterns linked to repeated wakefulness, abnormal REM sleep, and insomnia (Kim et al., 2019). While preliminary, such approaches provide quantifiable evidence to measure sleep as a risk factor for AD and may inform the development of sleep-based screening tools.

Olfactory dysfunction represents another behavioral domain with strong ties to AD pathology. Olfactory functioning involves multiple cognitive processes and coordinated neural systems, and disruption at any stage can result in olfactory dysfunction that predicts AD onset and progression (Liao et al., 2024). Olfactory dysfunction holds significant promise for detecting AD in the preclinical stage preceding MCI, and combining olfactory tests with other biomarkers may establish optimal models for AD prediction (Liao et al., 2024).

Simple clinical signs and brief questionnaires can also serve as potential first-line screening tools. The “head-turning sign” (HTS)—a patient’s behavior of turning toward their partner for assistance during an interview—and the three-question Neucop-Q (assessing consciousness/self-awareness of cognitive disabilities, pleasure/pastime, and news/knowledge on current topics) were validated against amyloid and tau PET in 155 patients (Daté et al., 2024). HTS positivity showed the highest specificity (0.930) and positive predictive value (0.870) for amyloid PET positivity, while impaired consciousness and impaired news/knowledge showed higher negative predictive values for amyloid PET negativity (0.750 and 0.725, respectively) (Daté et al., 2024). These simple screening tests were strongly associated with plasma Aβ42/40 ratio, pTau181, GFAP, and amyloid PET Centiloid, and may serve as potential first-line screening tools in memory clinics.

Repetitive negative thinking (RNT), a modifiable cognitive process common to psychological risk factors for AD, has also been linked to AD biomarkers. In 292 older adults from the PREVENT-AD cohort and 68 from the IMAP + cohort, RNT was associated with decline in global cognition, immediate and delayed memory, and global amyloid and entorhinal tau deposition, with relationships remaining after adjusting for potential confounders (Marchant et al., 2020). These findings suggest that modifying RNT may reduce AD risk, positioning behavioral and psychological markers as both diagnostic and interventional targets.

Wearable and portable technologies are also being developed to capture multiple behavioral data streams simultaneously. A wireless, portable soundscape monitoring system that combines auditory recording with EEG signal acquisition has been proposed for auditory profiling in high-risk populations, with the goal of providing a noninvasive diagnostic tool for early AD detection (Lam et al., 2020). While still in preliminary development, such systems illustrate the potential for continuous, home-based monitoring of behavioral and physiological biomarkers.

## 8 Clinical cognitive and functional assessment

Clinical cognitive assessment remains indispensable even when AD is defined biologically. Biomarkers identify pathological processes, whereas interview, cognitive testing, and functional assessment determine whether symptoms are present, which domains are affected, how impairment changes over time, and whether pathology plausibly explains the patient’s difficulties. The 2024 criteria therefore separate biological diagnosis from clinical staging and recognize that cognitive reserve, resistance, and copathology modify the relationship between biomarker burden and symptoms (Hazan et al., 2024; Jack et al., 2024).

Assessment should begin with a patient and informant history that establishes onset, tempo, domain-specific symptoms, and change from prior ability. Medication effects, delirium, sleep disorders, depression, sensory loss, vascular disease, and other reversible or contributing conditions must be considered. Informant evidence is particularly important when insight is reduced. Functional measures help distinguish objective cognitive impairment with preserved independence from dementia, in which cognition interferes with instrumental or basic daily activities. The Functional Activities Questionnaire, developed for community studies of normal aging and mild dementia, outperformed the Instrumental Activities of Daily Living Scale in separating normal from demented individuals.

Brief global screens are useful for case finding but should not be treated as stand-alone diagnostic tests. The MMSE is familiar and supports longitudinal comparison, yet it is relatively insensitive to mild impairment and executive dysfunction. The MoCA samples memory, executive, visuospatial, language, attention, and orientation domains and was developed to improve detection of MCI. In its original validation, at a cutoff of 26 the MoCA detected 90% of participants with mild cognitive impairment compared with 18% for the MMSE, although its specificity was lower (87% vs. 100%) (Nasreddine et al., 2005). Scores must be interpreted using appropriate language, education, age, cultural context, sensory status, and locally validated norms rather than a universal threshold.

Abnormal screening results, atypical presentations, high-functioning patients with persistent concerns, or discordance between symptoms and biomarkers warrant formal neuropsychological assessment. A domain-based battery should evaluate learning and delayed recall, recognition, attention and working memory, processing speed, executive control, language, visuospatial function, and social cognition when indicated. The pattern—not only the total score—can differentiate the typical amnestic syndrome from posterior cortical atrophy, logopenic aphasia, dysexecutive/behavioral AD, and non-AD disorders.

Clinical severity requires joint cognitive and functional staging. The Clinical Dementia Rating integrates memory, orientation, judgment and problem solving, community affairs, home and hobbies, and personal care to provide a global stage and sum-of-boxes measure (Morris, 1993). Repeated assessment should use consistent instruments and account for practice effects, measurement error, intercurrent illness, and meaningful change in everyday function. For preclinical and prevention research, composites such as the Preclinical Alzheimer Cognitive Composite combine episodic memory, executive function, and global cognition to detect subtle longitudinal decline (Donohue et al., 2014).

Cognitive results should guide, not merely follow, biomarker testing. We propose—as an expert framework awaiting prospective validation, not an evidence-based guideline—a stepped pathway (Figure 2): establish objective cognitive and functional change. Obtain structural imaging and routine laboratory evaluation; use blood-based biomarkers to refine AD probability where validated; and reserve CSF or PET for unresolved, atypical, or treatment-relevant cases. Of these steps, clinical assessment, structural imaging, and the use of validated blood biomarkers in specialized care have guideline support (Palmqvist et al., 2025). The exact sequencing, escalation thresholds, and handling of indeterminate results reflect expert interpretation and may reasonably vary with clinical setting, disease stage, and pretest probability. Concordant phenotype and biomarker findings strengthen etiological attribution. Discordance should prompt review of assay performance, disease stage, cognitive reserve, and alternative or mixed pathologies rather than automatic reclassification. Emerging remote and computerized tests may increase sensitivity and permit frequent assessment, but access, digital literacy, device effects, privacy, and algorithmic bias remain barriers. The central validation question is not whether a digital score correlates with amyloid or tau, but whether it detects clinically meaningful change and improves decisions across diverse real-world populations. Clinical cognitive assessment therefore provides the phenotypic anchor that converts a positive biomarker from a laboratory result into an interpretable diagnosis and stage.

**FIGURE 2:**
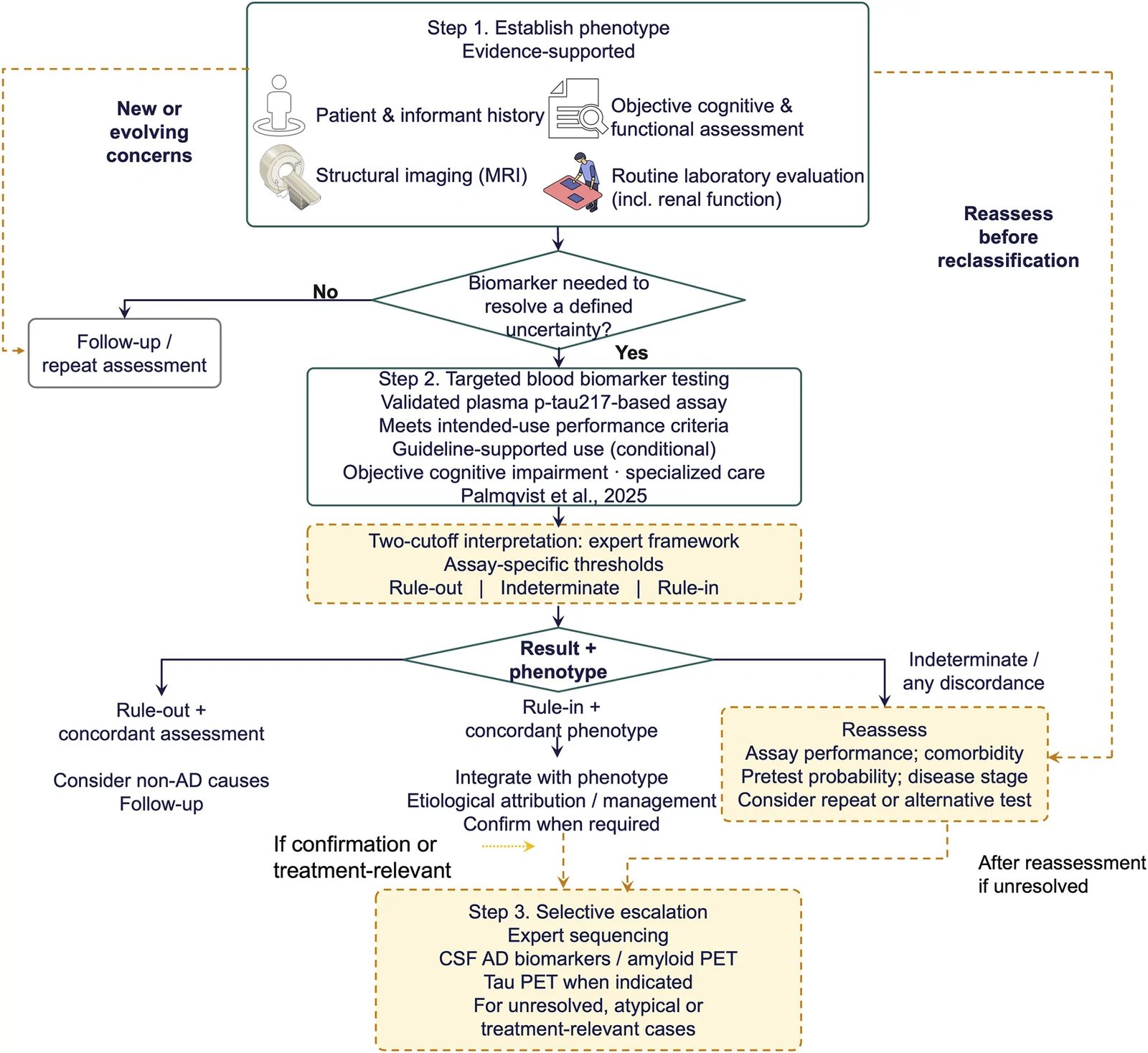
A stepped, phenotype-anchored pathway for the noninvasive diagnostic evaluation of suspected Alzheimer’s disease. Step 1 (phenotype establishment): patient and informant history, objective cognitive and functional assessment, structural imaging, and routine laboratory evaluation including renal function. Step 2 (targeted biological testing): validated blood biomarkers are added only when they resolve a defined diagnostic uncertainty, with assay-specific two-cutoff rule-in/rule-out interpretation and an indeterminate zone; indeterminate or discordant results loop back to reassessment rather than automatic reclassification. Step 3 (selective escalation): CSF or PET is reserved for unresolved, atypical, or treatment-relevant cases. Steps supported by current guidelines are distinguished from elements that reflect expert interpretation; the pathway is presented as an expert proposal awaiting prospective validation, and its application is expected to vary with clinical setting, disease stage, pretest probability, and comorbidity burden.

## 9 Multimodal integration and machine learning approaches

No single noninvasive measure captures the molecular cause, anatomical distribution, functional consequence, and clinical expression of AD. Multimodal approaches therefore combine complementary information: plasma p-tau and Aβ ratios estimate core pathology. MRI characterizes atrophy and vascular injury; PET resolves amyloid or tau distribution; cognitive and functional measures establish the phenotype; and digital or electrophysiological data permit repeated monitoring (Table 1).

A clinically useful architecture is sequential rather than indiscriminate. Low-cost history, cognitive assessment, routine testing, MRI, and validated blood biomarkers can define an initial probability. More expensive or less available tests should then be selected when they are likely to resolve a specific uncertainty or alter treatment. This strategy reduces unnecessary testing and avoids allowing a high-dimensional model to obscure discordance between biological and clinical evidence. This sequential architecture is consistent with recent professional guidance (Palmqvist et al., 2025), but no prospective study has yet compared it against alternative sequences with respect to patient outcomes.

Machine-learning studies show that combinations of plasma, imaging, genetic, and clinical features can improve discrimination or prognostic accuracy. Models using MRI radiomics and plasma indices predicted clinical conversion across two cohorts (Yu et al., 2024), while plasma, MRI, and clinical variables have been used to estimate tau-PET burden (Karlsson et al., 2025). Speech-derived measures combined with GFAP, p-tau217, APOE, and demographic data also improved classification of cognitive impairment and amyloid status (Wang et al., 2025). These examples support complementarity but do not establish universal superiority over a well-defined clinical pathway. The relevant benchmark is incremental clinical value over accessible assessments—clinician-led stepped evaluation or validated blood tests—assessed by calibration, decision-curve net benefit, and downstream testing or treatment impact, rather than by small AUC gains alone.

Newer fusion methods address missing or costly modalities. Cross-modal knowledge distillation can use paired MRI–PET data during training while requiring only MRI at inference (Wang et al., 2026), and joint synthesis-classification models can generate PET-like representations from MRI (Wang et al., 2024). Multiclass models that include MoCA scores alongside MRI and tau PET illustrate the contribution of cognitive data to separating normal cognition, MCI, and dementia (Huang et al., 2026). Synthetic modalities, however, remain predictions rather than substitutes for measured pathology and must be labeled accordingly.

For prognosis, longitudinal outcomes are more informative than cross-sectional case-control labels. A model combining plasma p-tau217, cognitive scores, and MRI or tau PET predicted trajectories on the Preclinical Alzheimer Cognitive Composite and improved simulated trial efficiency (Devanarayan et al., 2025b). Continuous disease models may also detect subtle change that categorical diagnoses miss (Tong et al., 2026). These approaches should be evaluated against clinically meaningful outcomes, including functional decline and treatment decisions.

Translation is limited by cohort selection, missing data, scanner and assay heterogeneity, demographic bias, data leakage, and weak external validation (Dehghani et al., 2024; Qi et al., 2025). Very high AUCs from small case-control datasets—particularly for EEG or imaging—often fall when tested across sites or against clinically relevant mimics. Validation should therefore include locked models, independent multicenter cohorts, calibration, subgroup performance, and prospective assessment in the intended clinical setting (Table 2).

Algorithmic fairness deserves specific attention. Machine-learning models for AD progression show unequal performance across sex, race, and ethnicity groups, with subgroup true-positive rates differing substantially even when aggregate accuracy appears acceptable (Yuan et al., 2023). Training cohorts that over-represent specific populations can lead models and fixed cutoffs to systematically misclassify under-represented groups; longitudinal biomarker trajectories differ by race (Xiong et al., 2022), and although middle-aged population data show broadly similar biomarker concentrations across race and ethnicity groups (Brickman et al., 2025), comorbidity distributions and assay-specific performance may still differ across populations. Validation should therefore report subgroup performance and fairness metrics alongside aggregate accuracy, and deployment should monitor whether errors concentrate in specific demographic groups.

Interpretability must extend beyond saliency maps. Clinicians need a report that identifies which measured variables drove the estimate, quantifies uncertainty, distinguishes missing from normal data, and shows whether the model is operating within its validated population. Decision-curve analysis and comparisons with clinician-led stepped evaluation are more informative than marginal improvements in discrimination.

The next-generation of multimodal systems should be designed around a defined clinical use case—screening, etiological confirmation, differential diagnosis, prognosis, or treatment monitoring. Success should be judged by added value over accessible cognitive, functional, blood, and imaging assessments; generalizability across populations; and demonstrable improvement in patient management rather than by benchmark accuracy alone.

## 10 Discussion and future perspectives

The field of AD diagnosis has shifted from a purely syndrome-based approach toward a biomarker-anchored framework (Dubois et al., 2016; Jack et al., 2016; Allegri, 2020). This transition enables detection of pathology before dementia, but it does not make clinical cognition dispensable. Biomarkers establish biological evidence; cognitive and functional assessment establish the phenotype, clinical stage, and consequences for the patient. The most useful noninvasive pathway therefore integrates these two forms of evidence rather than treating either as a standalone diagnosis.

The modalities reviewed here differ substantially in evidentiary maturity, and this hierarchy should guide interpretation. Plasma p-tau217 assays, amyloid PET, tau PET, and structural MRI rest on replicated multicenter validation, meta-analytic evidence, guideline endorsement, or regulatory clearance; retinal imaging, electrophysiology, sleep, speech, olfactory testing, and most machine-learning approaches remain largely exploratory, supported by single-cohort evidence with limited external validation. The descriptive maturity categories in Table 1 follow the definitions in the Methods. Biological plausibility, analytical validity, association with AD pathology, diagnostic accuracy, external validation, prospective clinical utility, and clinical readiness provide broader interpretive context for the strengths, limitations, and translational gaps of the evidence; these seven dimensions do not constitute a separate formal grading or scoring framework. Performance figures from exploratory modalities should therefore not be read as equivalent to those of validated modalities, and comparisons across maturity levels require matching populations and reference standards.

A central achievement in this field is the establishment of the A/T/N classification framework, which provides a standardized, biology-driven system for categorizing biomarker findings across amyloid (A), tau (T), and neurodegeneration (N) domains (Jack et al., 2016; Allegri, 2020). This system has enabled researchers to move beyond descriptive clinical labels and toward a molecular classification that aligns with the underlying proteinopathy (Allegri, 2020). The framework is agnostic to the temporal ordering of pathological events and does not presuppose a specific clinical diagnosis, making it suitable for population-based studies and clinical trials (Jack et al., 2016). However, the A/T/N system, while powerful, is not without limitations. It currently focuses on amyloid and tau pathology and neurodegeneration, and does not capture other co-pathologies such as TDP-43, α-synuclein, or vascular injury, which are common in older adults and may modify the clinical presentation (Hazan et al., 2024). Expanding the framework to include additional molecular targets will be necessary to achieve a comprehensive biological definition of AD and its mimics. Clinically, Lewy body disease, TDP-43 proteinopathy (LATE), and cerebrovascular injury can co-occur with AD and can themselves alter fluid and imaging markers—elevating NfL or GFAP, for example, or producing overlapping atrophy patterns—so biomarker results should be interpreted against the full pathological and clinical context rather than assumed AD-specific by default.

The push toward a purely biomarker-based definition of AD, as proposed in recent diagnostic criteria, has generated both enthusiasm and caution (Hazan et al., 2024). On one hand, a biological definition clarifies the entity of AD, which has historically been used interchangeably to describe both the neuropathological substrate and the clinical syndrome (Hazan et al., 2024). On the other hand, defining AD solely by amyloid and tau positivity risks a category error: it conflates the presence of pathology with the certainty of future clinical decline. Longitudinal data establishing the lifetime risk of dementia in amyloid-positive and tau-positive individuals remain incomplete, and the ethical implications of diagnosing asymptomatic individuals with a disease for which disease-modifying therapies are still limited must be carefully considered (Dubois et al., 2016; Hazan et al., 2024). For the present, the research domain remains the appropriate setting for biomarker-based diagnostic criteria, while the cautious adoption of plasma biomarkers in clinical practice may offer a pragmatic middle ground.

The accuracy of individual biomarkers has been systematically evaluated, and the evidence supports the clinical utility of several modalities. Amyloid PET and medial temporal lobe atrophy on MRI both demonstrate high sensitivity and specificity for neuropathologically defined AD, with median values exceeding 0.90 in well-conducted studies (Fink et al., 2020). However, the heterogeneity of study methods, the variability in biomarker cut points, and the uncertain applicability to typical clinical settings underscore the need for harmonization and standardization. The AD Neuroimaging Initiative has been instrumental in developing standardized protocols for MRI, PET, and CSF biomarkers across multicenter settings, and has demonstrated that multimodal classifiers combining imaging, fluid, and clinical data outperform any single modality (Weiner et al., 2015). These efforts must be extended to emerging biomarkers, including plasma p-tau217 and p-tau181, retinal imaging, and digital behavioral measures, to ensure that the same rigor applied to established biomarkers is applied to novel ones.

Looking forward, several priorities emerge for the next phase of research and clinical translation. First, the validation of biomarkers across diverse populations is essential. Most existing studies have been conducted in well-characterized academic cohorts with limited ethnic, racial, and socioeconomic diversity. The performance of biomarkers may differ in populations with varying comorbidity burdens, genetic backgrounds, and healthcare access. Large-scale, real-world studies that include primary care settings, memory clinics in underserved regions, and longitudinal community-based cohorts are needed to establish generalizability. Second, the integration of biomarkers into clinical workflows requires not only diagnostic accuracy but also practical considerations: cost, accessibility, patient acceptability, and the availability of trained personnel to interpret results. Plasma biomarkers, which are relatively low-cost and minimally invasive, are well-positioned for first-line screening. However, their performance in low-prevalence settings and their ability to reduce unnecessary downstream testing must be demonstrated in pragmatic trials (Hazan et al., 2024). Third, the role of biomarkers in therapeutic decision-making will become increasingly important as disease-modifying therapies enter clinical use. Updated appropriate use criteria for amyloid and tau PET provide guidance for selecting patients who are most likely to benefit from these treatments. However, the criteria must be revisited as new therapies and biomarkers emerge (Rabinovici et al., 2025).

The appropriate use criteria for amyloid and tau PET, updated in 2025, reflect the evolving clinical context. For amyloid PET, seven clinical scenarios were rated as appropriate, including the evaluation of patients with mild cognitive impairment or atypical dementia presentations when the diagnosis remains uncertain after standard evaluation (Rabinovici et al., 2025). For tau PET, five scenarios were rated as appropriate, reflecting its emerging role in staging disease severity and predicting progression (Rabinovici et al., 2025). However, many scenarios were rated as uncertain or rarely appropriate, highlighting that the clinical utility of these expensive and resource-intensive modalities is not yet fully established. The criteria emphasize that PET should be used judiciously, in conjunction with clinical evaluation and other biomarkers, and that its impact on patient outcomes must be systematically assessed.

The conceptual shift from neurodegenerative dementias to cognitive proteinopathies represents a deeper intellectual transformation in the field (Allegri, 2020). Clinical diagnosis, as traditionally practiced, tells us where abnormal tissue is found but not what molecular mechanisms are driving the pathology. Biomarkers allow us to answer the “what” question, linking specific proteinopathies to clinical phenotypes and enabling a precision medicine approach. This shift has implications beyond diagnosis: it informs the selection of participants for clinical trials targeting specific molecular pathways, guides the development of combination therapies for mixed pathologies, and provides a framework for understanding heterogeneity in disease progression. The challenge moving forward is to translate this conceptual advance into actionable clinical tools that can be deployed at scale, without losing sight of the patient experience and the ethical dimensions of early detection.

In summary, noninvasive AD diagnosis now spans molecular, structural, functional, cognitive, and digital domains. Blood biomarkers and imaging provide increasing biological specificity, while structured cognitive and functional assessment remains the anchor for clinical interpretation and longitudinal relevance. Electrophysiology is a promising complementary measure of network dysfunction, but currently has less direct etiological utility than validated fluid and imaging markers. Progress will depend on stepped clinical pathways, harmonized thresholds, representative prospective cohorts, and evidence that multimodal systems improve decisions and patient outcomes—not merely classification metrics.
